# S100A2 promotes clear cell renal cell carcinoma tumor metastasis through regulating GLUT2 expression

**DOI:** 10.1038/s41419-025-07418-1

**Published:** 2025-02-27

**Authors:** Mengli Deng, Shaoxia Liao, Jingwen Deng, Chen Li, Lu Liu, Qizheng Han, Yifeng Huo, Xiao Zhou, Xiaodong Teng, Maode Lai, Honghe Zhang, Chong Lai

**Affiliations:** 1 Department of Pathology, Zhejiang University School of Medicine, Research Unit of Intelligence Classification of Tumor Pathology and Precision Therapy, Chinese Academy of Medical Sciences (2019RU042), Hangzhou, 310058 Zhejiang China; 2 Department of Medical Oncology, Sir Run Run Shaw Hospital, Zhejiang University School of Medicine, Hangzhou, 310016 China; 3 Institute of Metabonomics & Medical NMR, School of Pharmaceutical Sciences, Wenzhou Medical University, Wenzhou, China; 4 Department of Pharmacology, China Pharmaceutical University, Nanjing, 210009 China; 5 Department of Pathology, the First Affiliated Hospital, Zhejiang University School of Medicine, Hangzhou, 310003 China; 6 Department of Urology, the First Affiliated Hospital, Zhejiang University School of Medicine, Hangzhou, 310003 China

**Keywords:** Metastasis, Oncogenesis

## Abstract

Clear cell renal cell carcinoma (ccRCC) is the predominant subtype of renal cancer and is highly malignant. Despite advances in diagnostics and treatment, the prognosis for ccRCC remains poor. The dual nature (promotion or inhibition) of S100A2 in different cancer types shows the complex involvement of its tumorigenesis, but its effect in ccRCC remains unclear. In this study, we first elucidate the tumor-promoting function of S100A2 in ccRCC by reprogramming glycolysis. Mechanistically, we demonstrate that S100A2 accelerates cancer progression through its interaction with the transcription factor HNF1A, leading to activating GLUT2 transcription. The upregulation of GLUT2 significantly enhances glucose uptake by cancer cells, thereby fueling augmented glucose metabolism and fostering the malignant progression of ccRCC. Collectively, our findings highlight the pivotal role of the S100A2-HNF1A-GLUT2 axis in promoting migration and invasion of ccRCC by amplifying glycolysis and suggest that targeting the S100A2-HNF1A-GLUT2 axis is clinically relevant for the treatment of metastatic ccRCC.

## Introduction

Renal cancer constitutes 2–3% of all adult cancers and results in approximately 102,000 global fatalities annually [1]. Clear cell renal cell carcinoma (ccRCC) stands out as the predominant histological subtype, comprising 75–80% of renal cancer cases [2]. Despite advancements in diagnostics and therapeutics, the prognosis for ccRCC patients remains suboptimal [3].

Glucose serves as the primary source for intracellular energy production and the generation of new cell mass. Even in the presence of ample oxygen, tumor cells exhibit a preference for glycolysis to metabolize glucose—a phenomenon recognized as the Warburg effect [4]. ccRCC is recognized as a metabolic disorder with significant alterations in various metabolic processes compared to normal kidney tissue [5]. Notably, glucose metabolism undergoes profound changes, emphasizing a crucial aspect of ccRCC’s metabolic shift [6, 7].

S100 proteins constitute a family of calcium-binding proteins with 25 family members, predominantly located on chromosome 1q21 [8]. The S100 protein family participates in various cellular processes—such as proliferation, migration, inflammation, and cell cycle regulation—that are essential for cancer initiation and progression [9]. Further investigation into their expression patterns and functional roles in ccRCC could uncover novel biomarkers, improving prognostic accuracy and potentially informing more personalized therapeutic approaches. The role of S100A2 in tumors is currently paradoxical. S100A2 demonstrates tumor-inhibiting properties, as observed in studies on liver cancer [10] and squamous cell carcinoma [11]. Conversely, in other malignancies such as non-small cell lung cancer [12], colorectal cancer [13], endometrial cancer [14], and pancreatic cancer [15], S100A2 appears to play a role in promoting tumor initiation and progression. The dual nature of S100A2 in different cancer types underscores the complexity of its involvement in tumorigenesis, suggesting that its function may be context-dependent. Further research is warranted to elucidate the precise mechanisms underlying the contradictory roles of S100A2 in various cancer contexts, providing valuable insights for potential therapeutic strategies.

This study first elucidates the role of S100A2 in ccRCC. S100A2 interacts with the transcription factor HNF1A to promote the transcription of the glucose transporter GLUT2. This interaction enhances the absorption and metabolism of intracellular glucose, consequently promoting the metastasis of clear cell renal cell carcinoma.

## Materials and methods

### Clinical materials

The patient cohort was from the First Affiliated Hospital, Zhejiang University School of Medicine, and was created to validate the decipher score as a predictor of developing metastatic disease. We collected 110 ccRCC tissues and assessed S100A2 expression levels using immunohistochemistry (IHC). All specimens were pathologically diagnosed by pathologists and IHC scoring was performed by two pathologists. All tissue samples were obtained from ccRCC patients who had not received any radiotherapy, chemotherapy, or other adjuvant therapies before surgery and diagnosis. This study was approved by the Institutional Ethics Committee of the First Affiliated Hospital, Zhejiang University School of Medicine (2021IIT923).

### Cell Culture and Treatment

Human clear cell renal cell carcinoma lines 769-P, 786-O, OSRC-2, and ACHN were cultured in Roswell Park Memorial Institute (RPMI) 1640 medium. The human HEK293T cell line was cultured in Dulbecco’s modified Eagle medium (DMEM). All media were supplemented with glutamine, 10% fetal bovine serum, and 1% penicillin/ streptomycin. Cell lines were grown in a humidified atmosphere at 37 °C with 5% CO_2_. 769-P, 786-O, OSRC-2, and ACHN were purchased from the American Type Culture Collection (ATCC, Manassas, VA, USA). HEK293T was purchased from the cell bank at the Chinese Academy of Sciences (Shanghai, China). All cells were undergone STR (Short Tandem Repeat) profiling analysis. For all the experiments, the cell lines with low passage numbers (<p15) were used. Mycoplasma was detected by Mycoplasma 156 kit (Shanghai Yeison Biotechnology). Plasmids were transfected into cell lines with LipoD293 (SignaGen). SiRNAs were transfected with GenMute siRNA Transfection Reagent (SignaGen).

### Transwell Migration and Invasion Assay

Cell motility and invasiveness were measured by transwell and matrigel chamber plates respectively (Corning Costar, USA). 1 × 10^5^ cells were loaded per transwell cultured with serum-free media in the upside of membrane and 10% fetal bovine serum was added at the bottom of the insert. After 20 h for 769-P and 786-O or 25 h for OSRC-2 and ACHN, the migrated cells were fixed in 4% paraformaldehyde (PFA) for 20 min and stained with crystal violet for another 5 min. Images were captured after air-drying, and the positive cells were quantified using the ImageJ software. Three fields of view were observed in each chamber and the experiment was repeated three times.

### Cell Proliferation Assays and Cell Viability Assays

Cell proliferation experiments were performed using Cell Counting Kit-8 (CCK-8) (Biosharp). A total of 2000 cells were seeded into each well of a 96-well plate and assayed at the same time every day. During the assay, 10 μL of the CCK-8 reagent was added to each well. The plate was incubated at 37 °C for 2 h before measuring the absorbance at 450 nm by collecting 80 μL of the solution. Each experimental group consisted of six replicate wells and the experiment was repeated three times.

### Colony Formation

For the short-term colony formation assay, cells were seeded in six-well plates (2000 cells per well) in RPMI 1640 medium and were cultured for 2 weeks. Then cells were fixed by paraformaldehyde (PFA) and stained with crystal violet in order to count cell numbers.

### Immunoblotting and immunoprecipitation

Whole cell lysates were extracted with RIPA lysis buffer (Fdbio Science) containing the protease inhibitor PMSF (Beyotime) followed by sonication. The supernatant was harvested by spinning at 13,300 rpm for 10 min at 4 °C. Protein concentrations were measured by bicinchoninic acid protein assay (Thermo Scientific). Equal amounts of protein lysates were separated by sodium dodecyl sulfate-polyacrylamide gel electrophoresis (SDS-PAGE). After transferring onto nitrocellulose membranes (Millipore), blocking with 5% milk and incubating with antibody overnight at 4 °C, followed by incubation with fluorescence secondary antibodies, detecting by Odyssey (LI-COR).

For co-IP, cells were lysed in lysis buffer (Beyotime) containing PMSF (Beyotime) and cocktail (MCE), followed by vortexing for 30 min, with 15 s of oscillations every 5 min. After centrifugation samples were rotated incubating with Anti-FLAG M2 Magnetic Beads (Sigma) or Anti-HA Magnetic Beads (Sigma) overnight at 4 °C and washed beads by wash buffer (Beyotime) several times, boiling samples in loading buffer with denaturant SDS. Equal amounts of total protein were immunoprecipitated with antibodies. Precipitates were analyzed by immunoblotting. An aliquot of each lysate was used as input control.

For endogenous co-IP assay, add the antibody to the pre-prepared cell lysate at the recommended antibody-to-protein ratio, and incubate overnight at 4 °C to allow sufficient binding of the antibody to the target protein. A Protein A/G immunoassay (coprecipitation) kit (Biolinkedin) was used to incubate with the antigen-antibody mixture for 3 h at room temperature. The mixture was washed by PBST three times and denatured by boiling with denaturant SDS.

### RT-qPCR

Total RNA was extracted with Trizol (Invitrogen) from the indicated cells. The cDNA was synthesized with HiScript II reverse transcriptase (Vazyme) and subjected to RT-qPCR with SYBR qPCR Master Mix (Vazyme) using gene-specific primers.

### Generation of S100A2 Knockout Cells

To achieve S100A2 knockout, two lentiviral packaging plasmids (psPAX2 and PMD2G) as well as pLentiCRISPR v2-S100A2 were cotransfected into HEK293T cells via lipoD293 according to the instructions and cell supernatants containing the virus were collected 48 h after transfection and infected with 769-P cell line for another 24 h. Thereafter puromycin was added to the culture medium. After one week, the remaining surviving cells were selected one by one into 96-well plates. These subclones were amplified and the knockout effect was checked by sequencing followed by immunoblotting. Clones that were not genomically edited were selected as mock cells.

### Mice Models for Metastasis

All animal experiments were performed in accordance with a protocol approved by the Institutional Animal Care and Use Committee at Zhejiang University.

Tail vein pulmonary metastasis in nude mice: OSRC-2 EV and S100A2-OE cell lines were injected into BALB/c nude mice (male, 6 weeks old) by tail vein. Based on a balance between statistical significance and experimental costs, we chose to experiment with 9 subjects in each group. We randomly divided the 18 mice into the EV group and S100A2-OE group, with 9 mice in each group. Each mouse was injected with 5 × 10^5^ cells suspended with 100 μL PBS. The mice were killed after 7 weeks and the lungs were removed for histological analysis.

Lung metastasis after orthotopic kidney injection in nude mice: OSRC-2 EV and S100A2-OE cell lines were injected into the kidney of BALB/c nude mice (male, 6 weeks of age) by performing surgery. Based on a balance between statistical significance and experimental costs, we chose to experiment with 8 subjects in each group. We randomly divided the 16 mice into the EV group and S100A2-OE group, with 8 mice in each group. The mice were killed after 8 weeks. After being killed, the lungs, kidneys, and other organs were separated. Lung and kidney were removed for histological analysis. If mice successfully receive injections of tumor cells via the tail vein and renal capsule, they are included in the study. All the mice are included.

### Hematoxylin and eosin staining

The mouse tissue samples were fixed in 4% paraformaldehyde and embedded in paraffin. Paraffin-embedded sections were deparaffinized and rehydrated. The sections were then stained with hematoxylin (Sigma) for 10 min. Subsequently, the sections were stained with eosin (Sigma) for a brief period of 2 s. Once the staining procedure was completed, the slides were scanned using a NanoZoomer digital slide scanner (Hamamatsu). Expert pathologists examined the tissue morphology, structures, and cellular morphology to discern any tissue alterations or changes.

### Immunohistochemistry

Paraffin-embedded clinical tissue samples were deparaffinized using xylene, followed by rehydration using a series of ethanol solutions of different concentrations. Endogenous peroxidase activity was blocked by treating sections with 3% hydrogen peroxide for 15 min at room temperature. To expose the antigens, a high-pressure treatment with 0.01 mol/L citrate buffer (ZSGB-BIO) was performed for 3 min. Sections were blocked by incubation with 10% bovine serum (Gibco) for 30 min at room temperature. The sections were then incubated overnight at 4 °C with the antibody. Following antibody incubation, the sections were incubated with goat anti-rabbit secondary antibody (ZSGB-BIO) for 30 min at room temperature. Finally, the sections were stained with 3,3’-diaminobenzidine (DAB) chromogen (ZSGB-BIO), counterstained with hematoxylin, dehydrated, coverslipped, and scanned using a NanoZoomer digital slide scanner (Hamamatsu).

### Immunofluorescence Assay

Cells were fixed in 4% paraformaldehyde for 20 min and then permeabilized in 0.1% Triton X-100 for 20 min. The cells were washed in PBS and then blocked with 10% normal goat serum for 30 min, followed by incubated antibodies overnight at 4 °C. The slides were then incubated with specific anti-Mouse Alexa 488 or anti-Rabbit Alexa 546 secondary fluorescence antibody for 1 h and then incubated with 4,6-diamino-2-phenyl indole (DAPI) (Thermo Fisher) for 20 min. Representative images of the pattern of location of each molecule are shown. All confocal analyses were repeated three times.

### Luciferase Reporter Assay

HEK293T cell line was co-transfected pGL3-basic or pGL3-GLUT2 and pcdna3.1, pcdna3.1-HNF1A or pcdna3.1-S100A2 with pRL-TK vector which was transfected as an internal reference. Cells were lysed and analyzed using the Dual-Luciferase Reporter Assay Kit (Promega) according to the manufacturer’s instructions 48 h after transfected.

### Measurement of Extracellular Acidification Rate

The Agilent Seahorse XF Glycolytic Rate Assay Kit (Agilent, USA) was used to determine the extracellular acidification rate (ECAR) according to the manufacturer’s instructions. Around 8 × 10^3^ cells per well were seeded in Seahorse XF cell culture plates overnight. The culture medium was replaced with the assay medium, and cells were incubated for 1 h at 37 °C in a CO_2_-free incubator before measurement. Cells were then sequentially treated with glucose, oligomycin, and 2-DG. The ECAR was measured using a Seahorse Wave (Agilent).

### Glucose Uptake

The rate of cellular glucose uptake was analyzed by the Glucose Assay Kit (Sigma-Aldrich, USA). Around 1 × 10^5^ cells per well were seeded in 24-well culture plates overnight. Cells were then cultured in 500 μL fresh medium for another 6 h and the culture medium set as the sample S1 was used for glucose measurement according to the manufacturer’s instructions. The fresh medium that was not used for cell culture was set as the sample S0. The absorbance of the samples was measured at 540 nm.

### Measurement of Lactate Secretion

The rate of cellular lactate secretion was analyzed using the Lactate Assay Kit (Nanjingjiancheng, China). Around 1 × 10^5^ cells per well were seeded in 24-well culture plates overnight. Cells were then cultured in 500 μL fresh medium for another 6–24 h. The culture medium was collected for lactate measurement and the absorbance of samples at 530 nm was determined according to the manufacturer’s instructions.

### NMR-based Metabolomic Analysis

The cells were collected and subjected to extraction using a methanol-chloroform-water method. Freeze-dried samples were reconstituted in 500 μL of D2O containing 0.04 mM sodium trimethylsilyl propionate-d4 (TSP) and transferred into 5-mm NMR tubes for analysis. The ¹H NMR spectra were recorded on a Bruker AVANCE III 600 MHz spectrometer equipped with a triple resonance probe at 298 K. A standard single-pulse sequence with water signal suppression (ZGPR) was employed, with acquisition settings as follows: 256 scans, 12,000 Hz spectral width, 64 K data points, 6 s relaxation delay, and 2.65 s acquisition time per scan.

Spectral preprocessing was carried out using Topspin (v2.1 pl4, Bruker Biospin, Germany), with the TSP signal (δ 0.0) used as a reference [16]. The spectrum range from 0.5 to 10.0 ppm was analyzed, excluding the water resonance region (4.8–5.1 ppm). For multivariate analysis, the spectrum was divided into 0.01 ppm intervals, while 0.0015 ppm intervals were used for quantitative assessment in MATLAB (R2012a, The Mathworks Inc, Natick, MA, USA) [17]. Metabolite concentrations were calculated based on peak areas relative to TSP and expressed as relative units (r.u.). Changes in metabolic profiles were assessed through normalized integral values. Specifically, after reducing the spectral data, segment integrals were normalized to the total sum of all integrals. Each segment contained the signal and area of specific metabolites. Statistical tests were then applied to these normalized integrals to identify differences between experimental groups. This approach is widely recognized and has been implemented in prior studies [18, 19].

### TCGA Database Analysis

Using the TCGA (The Cancer Genome Atlas) database, we analyzed the ccRCC patient dataset to investigate the impact of S100 genes expression on patient survival outcomes. We stratified the patients into two groups based on the median expression level of S100: a high S100 expression group and a low S100 expression group. The log-rank test was employed to determine the statistical significance of the differences observed in survival curves.

### RNA-seq and Enrichment Analysis

PolyA-RNA of 769-P-MOCK, 769-P-KO, OSRC-2-EV, and OSRC-2-OE cells were extracted, sequenced, and analyzed by Bioacme (Wuhan, China). Three biological replicates were used for the condition. The individual RNA seq libraries were pooled based on their respective sample specific-6 bp adaptors and sequenced at 150 bp/sequence pair-read using an Illumina NovaSeq system. Reads were mapped into the hg19 reference genome by STAR50 and quantified by RSEM51. Gene differential expression analysis was accomplished by the DESeq252 package in R. Benjamini–Hochberg false discovery rate method was applied to correct for multiple hypothesis testing. The genes with adjust *p* < 0.05, fold change >2, or <0.5 were defined as different expression genes (DEGs) as candidates for further analysis. Gene expression heatmap was accomplished with R package heatmap. We performed KEGG enrichment analysis using the R package “clusterProfiler”, considering KEGG terms with a *P* value < 0.05 as statistically significant. The results were visualized using the ‘ggplot2’ R package.

### Quantifications and Statistical Analysis

The statistical tests are described in the figure legends. The data in this paper were expressed as mean ± SD, and an unpaired t-test was used to compare the mean differences between two independent samples. One-way ANOVA compares mean differences across three or more groups for a single factor, while two-way ANOVA analyzes the effects of two factors on the dependent variable, including their interaction. Kaplan-Meier survival analysis was performed using the software IBM SPSS Statistics 20, using the Log-rank test. *p* value < 0.05 was considered statistically significant, *p < 0.05, **p < 0.01, ***p < 0.001, ****p < 0.0001, NS, not significant.

## Results

### High expression of S100A2 is associated with poor prognosis in ccRCC patients

**Fig. 1:**
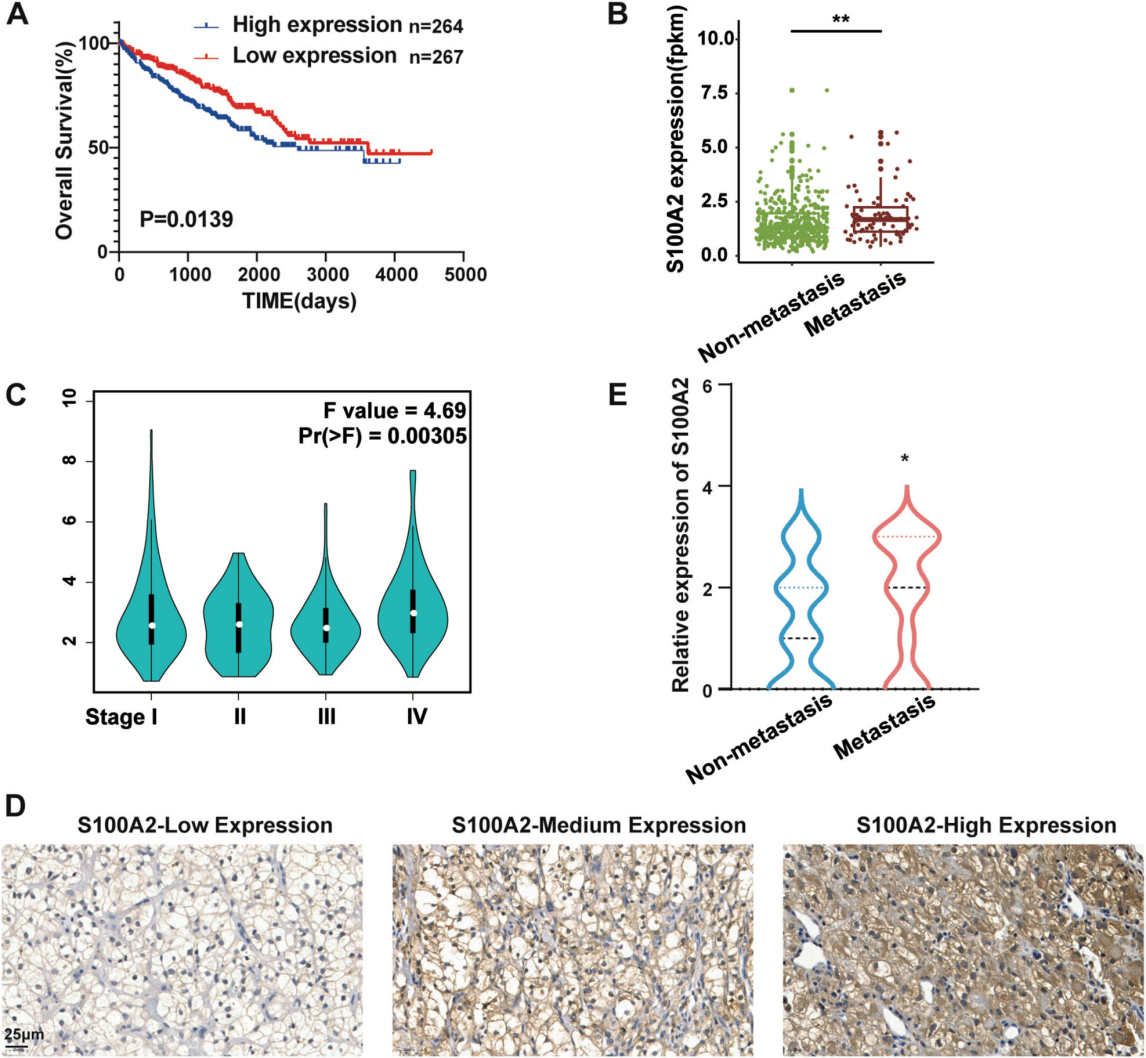
High expression of S100A2 is associated with poor prognosis in ccRCC patients. **A** Overall survival’s Kaplan-Meier curves based on S100A2 expression in ccRCC samples from TCGA datasets. **B** Expression of S100A2 in non-metastasis and metastasis ccRCC samples from TCGA datasets. **C** S100A2 expression in ccRCC progression stages I-IV from TCGA datasets. **D** Photomicrographs showing the staining intensity of S100A2 protein by immunohistochemistry. **E** Expression of S100A2 in non-metastasis and metastasis ccRCC samples from our cohort. Statistical analyses were performed using the log-rank test (**A**), the Wilcox rank test (**B**, **E**), and the one-way ANOVA (**C**). *, *p* < 0.05, **, *p* < 0.01. ***p < 0.001, ****p < 0.0001.

To elucidate the clinical significance of the S100 family in ccRCC, we used the TCGA database to conduct a survival analysis. Patients were divided into high-expression and low-expression groups based on the median S100 expression level in ccRCC tumors. Among these proteins, elevated S100A2 and S100A11 expression emerged as pivotal factors, correlating significantly with poorer prognosis in ccRCC (Fig. 1A and Supplementary Fig. 1A). Extensive research has already clarified the biological roles and molecular mechanisms of S100A11 in ccRCC [20, 21], prompting us to concentrate our study on S100A2 instead. We further confirmed the association between high S100A2 expression and patient prognosis using the renal cancer dataset (E-DKFZ-1) from the ArrayExpress database (Supplementary Fig. 1B). A closer examination revealed heightened S100A2 expression in patients with metastasis compared to those without metastasis (Fig. 1B). Analyzing the correlation between S100A2 mRNA expression and ccRCC stage in the TCGA dataset via GEPIA 2 website indicated an upregulation of S100A2 at the advanced tumor stage (Fig. 1C) [22]. To further determine the clinical significance of S100A2, we collected tumor samples from 110 ccRCC patients. Using immunohistochemical scoring, we categorized the samples into low, medium, and high S100A2 expression levels (Fig. 1D). S100A2 expression was higher in metastatic samples (Fig. 1E). As shown in Table 1, high expression of S100A2 was significantly correlated with metastasis (*p* = 0.004634), and TNM stage (*p* = 0.03957), while there was no prominent association of S100A2 expression with age, gender, and primary site. These findings collectively suggest that S100A2 may play a pivotal role in promoting tumor progression during the later stages of ccRCC development.

**Table 1:** The correlation of clinicopathological variables of the ccRCC patients and S100A2 expression level.

Clinicopathological	Cases	S100A2 Level			X^2^	*P* value
Parameters		High (32)	Medium (28)	Low (50)		
**Age**	106				1.6483	0.4386
<60	59	19	16	24		
≥60	47	12	10	25		
**Gender**	108				0.62278	0.7324
Female	34	11	7	16		
Male	74	20	20	34		
**Primary site**	103				0.93236	0.9199
Upper Lobe	38	11	8	19		
Middle Lobe	25	7	6	12		
Lower Lobe	40	13	11	16		
**Metastasis**	110				10.749	0.004634
Yes	57	24	14	19		
No	53	8	14	31		
**TNM Stage**	109				6.4591	0.03957
I- II	51	9	14	28		
III -IV	58	23	13	22		

### S100A2 promotes migration and invasion in vitro

**Fig. 2:**
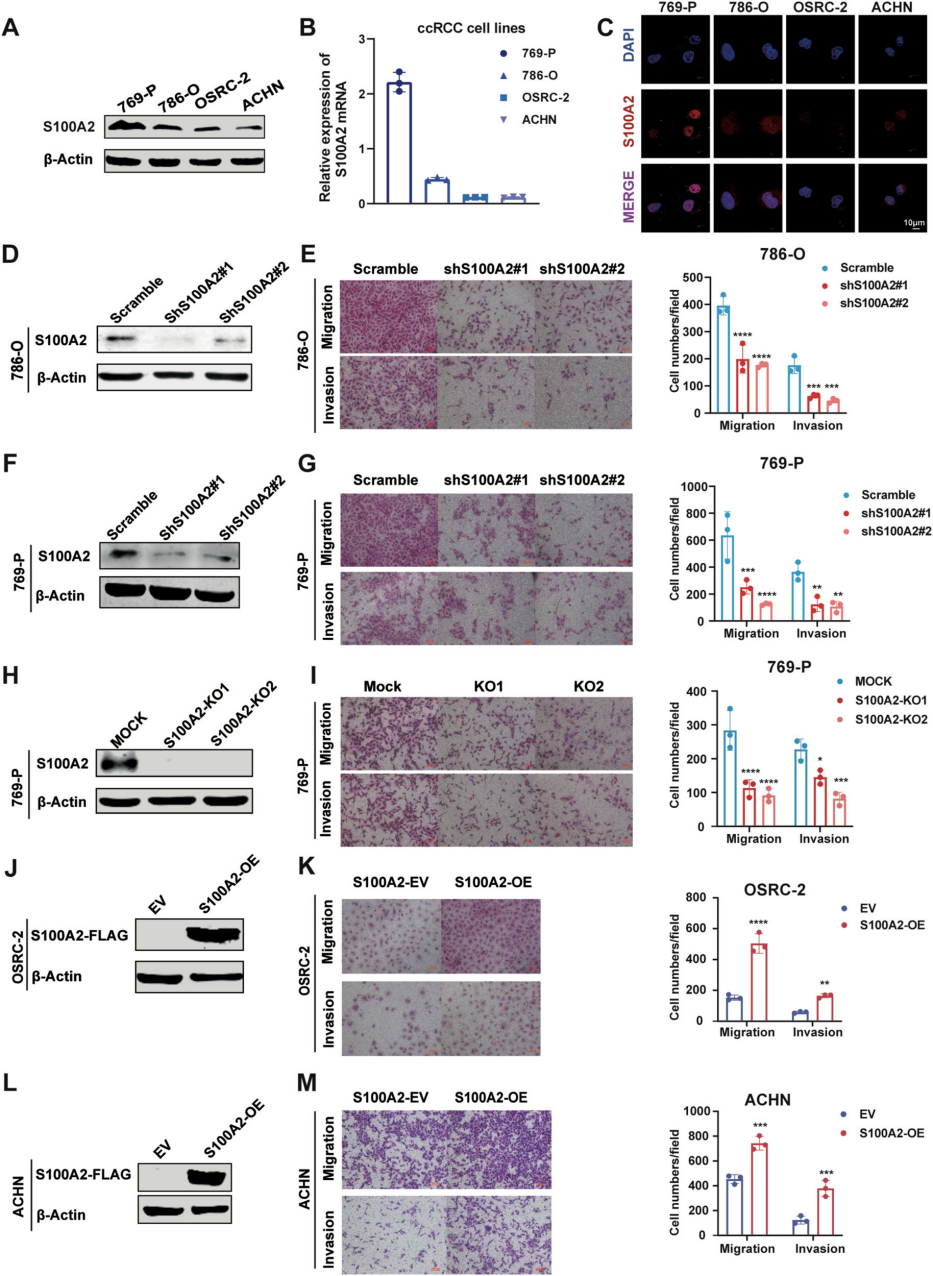
S100A2 promotes migration and invasion in vitro. **A** Western blot analysis of S100A2 protein expression in 769-P, 786-O, OSRC-2, and ACHN cells. **B** Real-time qPCR analysis of S100A2 mRNA expression in several ccRCC cell lines. **C** Immunofluorescence stain analysis of the subcellular localization of S100A2 (red) in several ccRCC cell lines. **D** Western blot analysis of S100A2 in 786-O cells with knocking down of S100A2 by shRNA. **E** Transwell assay for evaluating the migration and invasion abilities of S100A2 knockdown 786-O cells. The histograms on the right show the quantitative analysis results. **F** Western blot analysis of S100A2 in 769-P cells with knocking down of S100A2 by shRNA. **G** Transwell assay for evaluating the migration and invasion abilities of S100A2 knockdown 769-P cells. The histograms on the right show the quantitative analysis results. **H** Western blot analysis of S100A2 proteins in 769-P cells with knocking out of S100A2 by CRISPR-Cas9. **I** Transwell assay for evaluating the migration and invasion abilities of S100A2 knockout 769-P cells. The histograms on the right show the quantitative analysis results. **J** Western blot analysis of S100A2 protein overexpression in OSRC-2 cells. **K** Transwell assay for evaluating the migration and invasion abilities of S100A2-overexpression OSRC-2 cells. The histograms on the right show the quantitative analysis results. **L** Western blot analysis of S100A2 protein overexpression in ACHN cells. **M** Transwell assay for evaluating the migration and invasion abilities of S100A2-overexpression ACHN cells. The histograms on the right show the quantitative analysis results. The data are presented as the mean ± SD values; statistical significance was assessed by two-way ANOVA. *P < 0.05, **P < 0.01, ***P < 0.001, ****P < 0.0001.

To investigate the function of S100A2 in ccRCC, we detected the expression of S100A2 at both the protein and mRNA levels in four distinct ccRCC cell lines (Fig. 2A, B). Notably, 769-P and 786-O cells exhibited relatively high S100A2 expression. Employing immunofluorescence experiments, we determined the subcellular localization of S100A2 in ccRCC, revealing its predominant presence in the nucleus and cytoplasm of cancer cells (Fig. 2C). First, we used shRNAs to knock down S100A2 in 786-O (Fig. 2D and Supplementary Fig. 2A) and 769-P cells (Fig. 2F and Supplementary Fig. 2D). CCK8 and colony formation assay revealed that S100A2 downregulation did not influence cell proliferation (Supplementary Fig. 2B-C and Supplementary Fig. 2E-F). However, the transwell assay results showed that the migration and invasion ability was decreased in both cell lines when S100A2 was knocked down (Fig. 2E, G). Further, we used CRISPR-Cas9 to knock out S100A2 in the 769-P cell line (Supplementary Fig. 2G and Fig. 2H). The migration and invasion potential were significantly decreased (Fig. 2I), while cell proliferation remained unaffected (Supplementary Fig. 2H and I). Consistent with these results, overexpression of S100A2 in S100A2-knockout 769-P cells, the migratory and invasive phenotype was restored (Supplementary Fig. 2J and K). Furthermore, overexpression of S100A2 in OSRC-2 (Fig. 2J) and ACHN (Fig. 2L) promotes cell migration and invasion (Fig. 2K, M). Remarkably, this effect on migration and invasion did not extend to cell proliferation, as evidenced by the CCK-8 assay and plate cloning assay (Supplementary Fig. 2,L-O). Additionally, we found that the migratory and invasive abilities of OSRC-2 cells increased with elevated expression of S100A2(Supplementary Fig. 2P and Q). Meanwhile, when siRNAs were used to knock down S100A2 in S100A2-overexpressing cells, the migratory and invasive phenotype was restored (Supplementary Fig. 2R and S). Collectively, these results confirmed the role of S100A2 in promoting migration and invasion in ccRCC cell lines.

### S100A2 promotes tumor metastasis in vivo

**Fig. 3:**
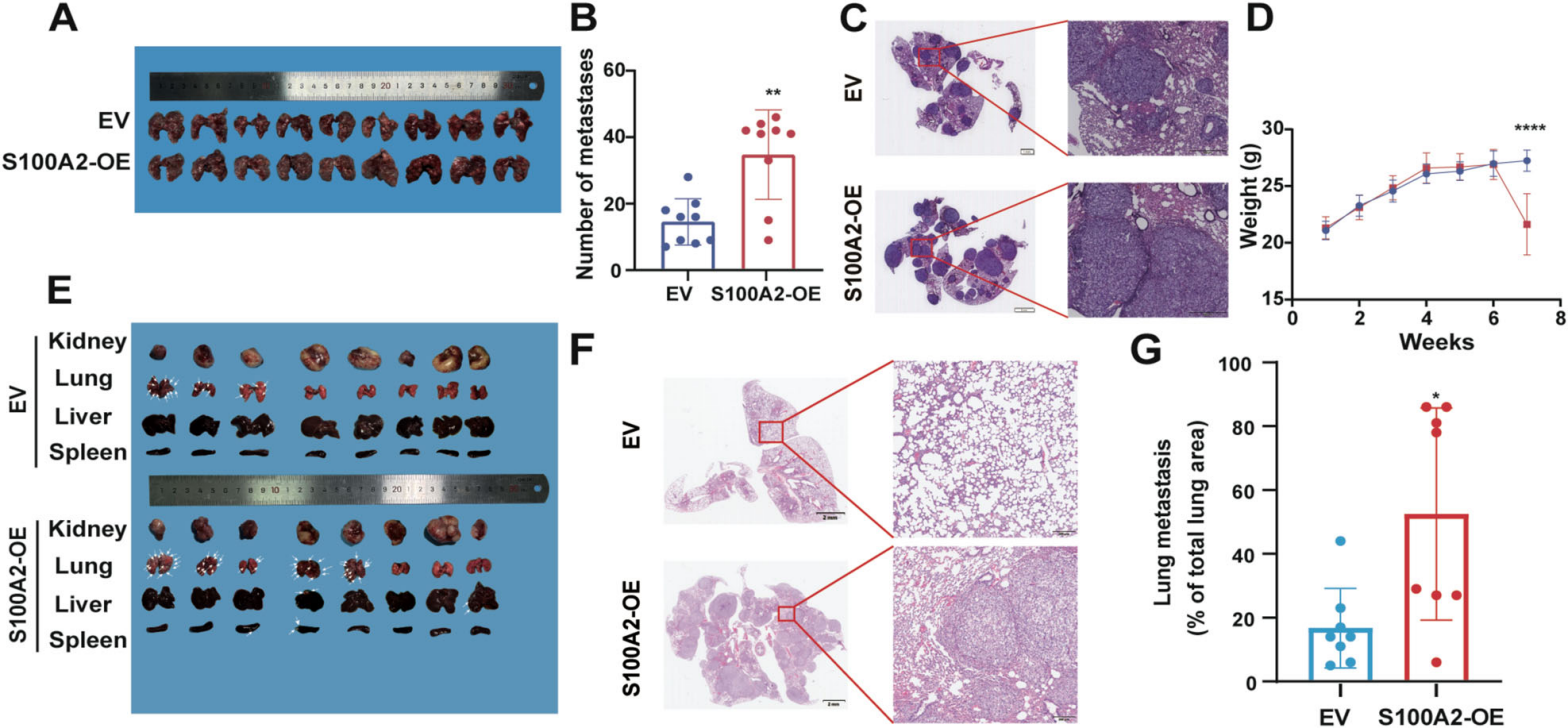
S100A2 promotes tumor metastasis in vivo. **A** Images of lung distant metastatic lesions. **B** Number of lung distant metastatic lesions. **C** H&E staining of lung metastatic foci. **D** Body weight of nude mice after tail vein injection. **E** Images of distant metastatic lesions. **F** H&E staining of lung metastatic foci. **G** Lung metastatic burden as quantified by H&E staining. The data are presented as the mean ± SD values; statistical significance was assessed by unpaired t-test. *P < 0.05, **P < 0.01, ***P < 0.001, ****P < 0.0001.

To investigate whether S100A2 promotes ccRCC metastasis in vivo, OSRC-2 cells with either empty vector (EV) or S100A2 overexpression(S100A2-OE) were intravenously injected into BALB/c mice by tail vein. Seven weeks later, the S100A2-OE group exhibited a higher number of metastatic tumors in lung tissues (Fig. 3A, B). HE staining also revealed more pronounced tumor infiltration in the lung tissue of the S100A2-OE group (Fig. 3C). Furthermore, body weight loss was observed in the S100A2-OE group compared to the EV group (Fig. 3D). To further elucidate the role of S100A2 in ccRCC metastasis in vivo, we injected EV or S100A2-OE OSRC-2 cells into the renal sub capsule of BALB/c mice. After 8 weeks, the S100A2-OE group exhibited more metastatic foci (Fig. 3E). In the EV group, metastasis primarily occurred in the lung tissue, whereas the S100A2-OE group exhibited metastasis in the liver and spleen as well. As confirmed by HE staining, which also revealed more pronounced tumor infiltration in the lung tissue of the S100A2-overexpressed group (Fig. 3F, G). These results indicate that S100A2 plays a role in promoting tumor metastasis in vivo.

### S100A2 regulates the glycolysis pathway in ccRCC

**Fig. 4:**
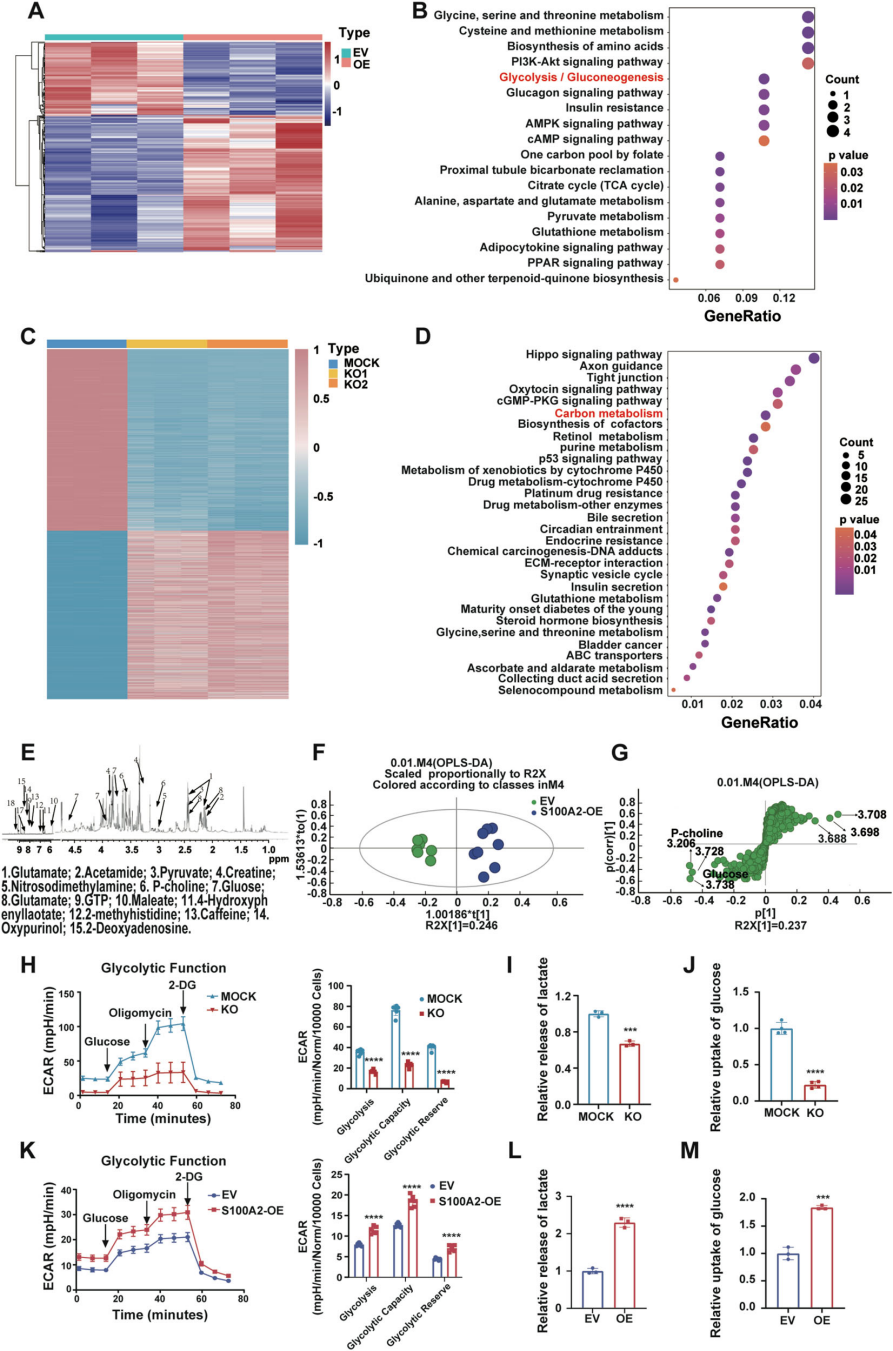
S100A2 promotes ccRCC migration by regulating the glycolysis pathway. **A** Heat map of DEGs in OSRC-2 EV versus S100A2-OE cells identified via RNA-seq. **B** Kyoto Encyclopedia of Genes and Genomes of RNA-seq data from EV and S100A2-OE OSRC-2 cells. **C** Heat map of DEGs in 769-P mock versus S100A2-KO1 and S100A2-KO2 cells identified via RNA-seq. **D** Kyoto Encyclopedia of Genes and Genomes of RNA-seq data from 769-P mock versus S100A2-KO1 and S100A2-KO2 cells. **E** Typical ^1^H-NMR spectra obtained from OSRC-2 EV and S100A2-OE cells. **F** Loading plots of PCA analysis of indicated cells based on ^1^H-NMR data. **G** Score plots of PCA analysis from OSRC-2 EV and S100A2-OE cells. **H** Glycolytic function assay in 769-P mock versus S100A2-KO cells identified via ECAR assay, quantification of glycolysis, glycolytic capacity, and glycolytic reserve in the ECAR assays. **I** Lactate production analysis of the mock and S100A2-KO 769-P cells. **J** Glucose uptake analysis of the mock and S100A2-KO 769-P cells. **K** Glycolytic function assay in OSRC-2 EV versus S100A2-OE cells identified via ECAR assay, quantification of glycolysis, glycolytic capacity, and glycolytic reserve in the ECAR assays. **L** Lactate production analysis of the EV and S100A2-OE OSRC-2 cells. **M** Glucose uptake analysis of the EV and S100A2-OE OSRC-2 cells. The data are presented as the mean ± SD values; statistical significance was assessed by two-way ANOVA (**H** and **K**), and unpaired t-test (**I**, **J**, **L**, and M). *P < 0.05, **P < 0.01, ***P < 0.001, ****P < 0.0001.

To explore the potential molecular mechanisms underlying the promotion of ccRCC metastasis by S100A2, we conducted bulk-RNA sequencing in the S100A2-OE OSRC-2 cell line. The S100A2-OE cells exhibited 832 differentially expressed genes (DEGs) (Fig. 4A). We identified the top upregulated differentially expressed genes (DEGs) in S100A2-overexpressed cell lines, with enrichment in the glucose metabolism pathway via Kyoto Encyclopedia of Genes and Genomes (KEGG) analysis (Fig. 4B). Additionally, a bulk-RNA sequencing on two S100A2-knockout (KO) 769-P cell lines revealed 4111 overlapping DEGs, enriched in the carbon metabolism pathway according to KEGG analysis (Fig. 4C, D). To validate these DEGs, we investigated the expression of glycolysis pathway-related genes (ENO2, GPI, NUP58, SLC37A1, PKLR, and PFKFB3) using RT-qPCR (Supplementary Fig. 3A).

Based on the hypothesis that S100A2 influences glucose metabolism to promote tumor metastasis, we performed ^1^H-NMR-based metabolomics on OSRC-2 cells. The ^1^H-NMR spectra revealed metabolites associated with glycolysis and other pathways (Fig. 4E). Utilizing Orthogonal Partial Least Squares-Discriminant Analysis (OPLS-DA), S100A2-OE OSRC-2 cells were distinguished from EV cells based on the first principal component (Fig. 4F). The S-plot of OPLS-DA highlighted significant metabolites, such as phospholipids and glucose (Fig. 4G), with glucose being significantly up-regulated in the S100A2-OE group compared to the EV group (Supplementary Fig. 3B).

Then, we investigated the underlying reprogrammed glucose metabolism modulated by S100A2. To assess the influence of S100A2 on glycolysis, the extracellular acidification rate (ECAR) was measured. Through ECAR, we observed that the level of glycolysis was decreased by the depletion of S100A2 in 769-P cells, while increased by S100A2 overexpression in OSRC-2 cells (Fig. 4H, K). To further explore the role of S100A2 on glucose metabolism in ccRCC, we detect the capacity of glucose uptake and lactate production. We found that knockout S100A2 decreases the lactate production capacity in 769-P cells (Fig. 4I), and overexpression of S100A2 enhances the lactate production capacity in OSRC-2 cells (Fig. 4L). Meanwhile, the glucose uptake assays demonstrated that knockout S100A2 decreases the absorption capacity in 769-P cells (Fig. 4J), and overexpression of S100A2 enhances the absorption capacity in OSRC-2 cells (Fig. 4M). Consistently, overexpression of S100A2 in S100A2-knockout 769-P cells, the level of glycolysis, the glucose uptake phenotype, and lactate production phenotype were restored (Supplementary Fig. 3C-E). Further, when siRNAs were used to knock down S100A2 in S100A2-overexpressing cells, the level of glycolysis, the glucose uptake phenotype, and lactate production phenotype were restored (Supplementary Fig. 3F–G). To further verify changes in glucose metabolism are the direct reason the S100A2 overexpression promotes metastasis, we treated OSRC-2 S100A2-OE cell line with the glucose inhibitor 2-DG and found that 2-DG inhibited the enhanced invasion and migration induced by S100A2 overexpression in dose-dependent manner (Supplementary Fig. 3I). Taken together, these data underscore the crucial role of S100A2 in regulating the Warburg effect, a metabolic shift associated with cancer aggressiveness.

### S100A2 enhances the Warburg Effect by regulating the expression of GLUT2

**Fig. 5:**
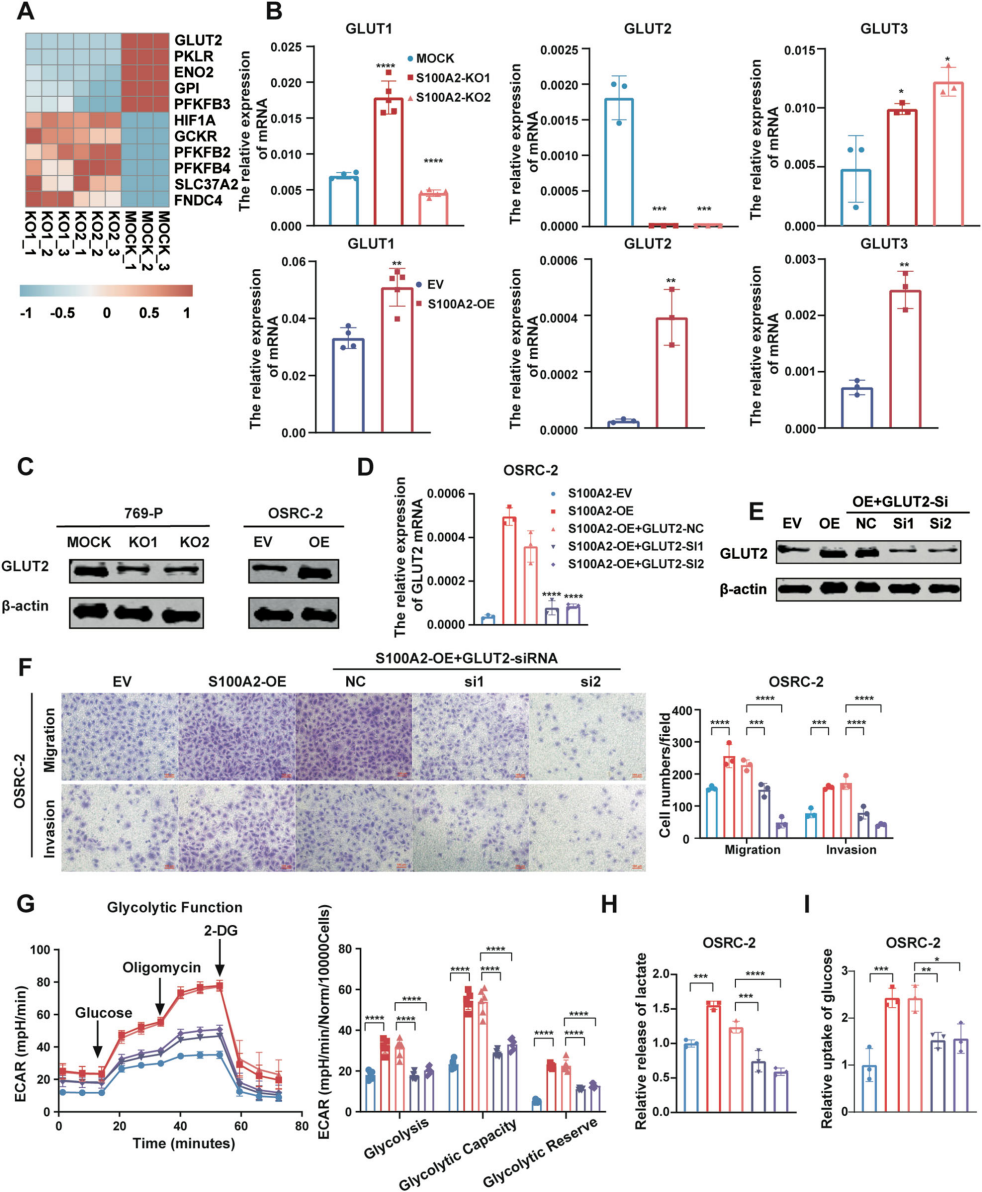
S100A2 enhances the Warburg Effect by regulating the expression of GLUT2. **A** Heatmap of glycolysis-related genes regulated by S100A2. **B** RT-qPCR analysis of the relative mRNA expression of the glucose transporters, including GLUT1, GLUT2, and GLUT3. **C** Immunoblotting assay for expression of GLUT2 in OSRC-2 and 769-P cells with S100A2 overexpression or knockout. **D** RT-qPCR analysis of GLUT2 mRNA expression in S100A2-overexpression and re-silencing GLUT2 in OSRC-2 cells. **E** Western blot analysis of GLUT2 protein expression in S100A2-overexpression and re-silencing GLUT2 in OSRC-2 cells. **F** Transwell assay for evaluating the migration and invasion of OSRC-2 cells with S100A2-overexpression and re-silencing GLUT2 in OSRC-2 cells. The histograms on the right show the quantitative analysis results. **G** ECAR assay for evaluating the glycolytic function of OSRC-2 cells with S100A2-overexpression and re-silencing GLUT2 in OSRC-2 cells. The histograms on the right show the quantification of glycolysis, glycolytic capacity, and glycolytic reserve in the ECAR assays. **H** Lactate excretion of EV, S100A2-OE, and GLUT2-si in S100A2-OE OSRC-2 cells. **I** Glucose uptake of EV, S100A2-OE, and GLUT2-si in S100A2-OE OSRC-2 cells. The data are presented as the mean ± SD; statistical significance was assessed by an unpaired t-test (**B**), one-way ANOVA (**D**, **H**, **I**), and two-way ANOVA (**F**, **G**). (*P < 0.05, **P < 0.01, ***P < 0.001, ****P < 0.0001.

In order to investigate the role of S100A2 in glucose metabolism, we conducted a further analysis of the genes related to the glycolysis pathway in the RNA-seq data. Among them, GLUT2 showed significant differential expression (Fig. 5A). GLUT2, also known as SLC2A2, is a member of the glucose transporter family. Cells depend on glucose transporters on their surface for glucose uptake, and there are 14 kinds of GLUTs in the human body, with the most well-understood ones being GLUT1,2,3,4 [23]. We test the expression of glucose transporter proteins in 769-P cells using MOCK and S100A2-KO constructs, as well as OSRC-2 cells with S100A2-OE constructs. Among these cell lines, GLUT4 was almost not expressed. The mRNA expression of GLUT2 was found to be reduced in S100A2-KO cells compared to S100A2-MOCK cells. Moreover, the expression level of GLUT2 was found to be higher in S100A2-OE cells than in EV cells (Fig. 5B). Western blot experiments revealed a similar result that S100A2 knock-out inhibited and S100A2 overexpression promoted GLUT2 expression (Fig. 5C). To further confirm that S100A2 regulates the glycolysis level of ccRCC cells by GLUT2, we knocked down GLUT2 in the S100A2-OE cell line (Fig. 5D, E). Knock-down of GLUT2 predominantly aborted the enhancement of cell migration and invasion after S100A2 overexpression (Fig. 5F). Consistent with these results, when siRNAs were used to silence GLUT2 in S100A2-overexpressing cells, the phenotype of ECAR (Fig. 5G), lactate production (Fig. 5H), and glucose absorption (Fig. 5I) were restored.

In the majority of renal clear cell carcinoma cases, the activity of the von Hippel-Lindau tumor suppressor (VHL) protein is lost due to genetic or epigenetic changes in the cancer cell genome. The loss of the VHL function leads to a gain of the HIF-1α function. HIF-1α is essential for tumorigenesis in VHL-deficient renal carcinoma cells. HIF-1α plays a critical role in glucose metabolism [24]. To investigate the effect of S100A2 on glucose metabolism, we examined its impact on HIF-1α in the VHL-deficient cell line OSRC-2. The results showed that S100A2 overexpression did not affect the expression levels of HIF1α (Supplementary Fig. 4A). Further, we detected in ACHN cell lines with normal VHL expression and found that S100A2 still affected GLUT2 expression (Supplementary Fig. 4B), which indicates that the regulation of GLUT2 by S100A2 is not affected by VHL-HIF1α. Collectively, these results illuminate that S100A2 affects the glycolytic capacity of ccRCC through GLUT2.

### S100A2 interacts with HNF1A to promote transcriptional regulation of GLUT2

**Fig. 6:**
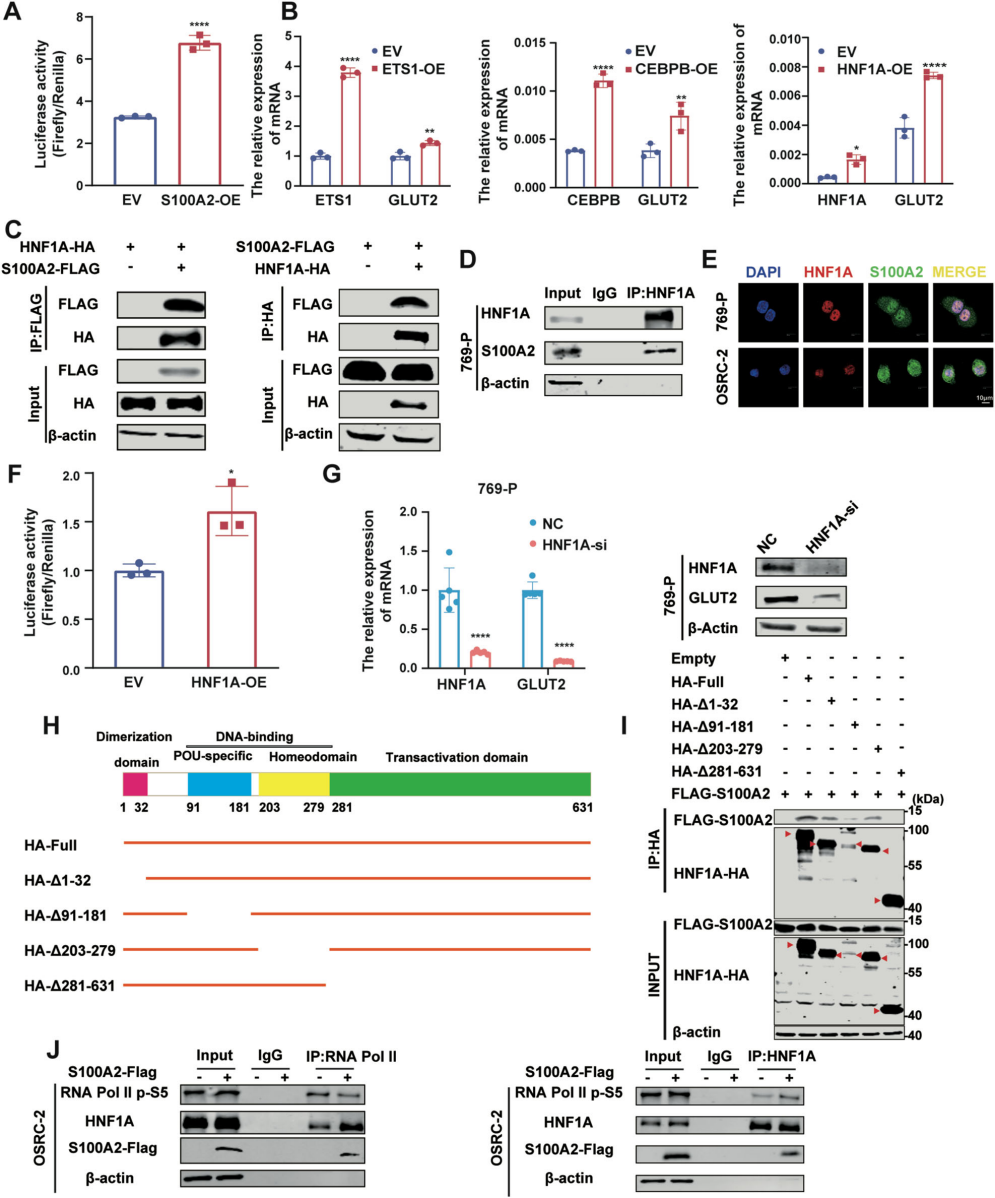
S100A2 overexpression increases GLUT2 expression by interacting with HNF1A. **A** Luciferase reporter assay of GLUT2 promoter activity in EV and S100A2-OE cells. **B** RT-qPCR analysis of GLUT2, ETS1, CEBPB, and HNF1A mRNA expression in ETS1, CEBPB, and HNF1A overexpression cells. **C** Western blot analysis to detect IP of exogenous FLAG-tagged S100A2 and HA-tagged HNF1A by an anti-Flag antibody or by an anti-HA antibody in HEK293T cells. **D** Western blot analysis to detect IP of endogenous S100A2 and HNF1A by an anti-HNF1A antibody in 769-P cells. **E** Confocal microscopy and immunofluorescence staining revealed that S100A2 and HNF1A colocalize in the nucleus. **F** Luciferase reporter assay of GLUT2 promoter activity in EV and HNF1A-overexpressing cells. **G** RT-qPCR and western blot analysis of GLUT2 expression while knocking down HNF1A. **H** Schematic of the functional domains of HNF1A and the deletion constructs. **I** Immunoblotting to detect the immunoprecipitation of exogenous Flag-tagged S100A2 and HA-tagged HNF1A with the full-length sequence or deletion mutations by an anti-HA antibody in HEK293T cells. **J** Western blot analysis to detect IP of endogenous RNA Pol II and HNF1A by an anti-RNA Pol II antibody or by an anti-HNF1A antibody in EV and S100A2-OE OSRC-2 cells. The data are presented as the mean ± SD; statistical significance was assessed by an unpaired t-test (**A,** **F**), and two-way ANOVA (**B**, **G**). *P < 0.05, **P < 0.01, ***P < 0.001, ****P < 0.0001.

To further investigate how S100A2 regulates GLUT2, we performed a luciferase assay, which showed that S100A2 over-expression was associated with enhanced promoter activity of GLUT2 (Fig. 6A). Previous studies have reported that S100A2 can act as a cofactor and interact with transcription factors to promote their transcriptional activity [13]. Therefore, we searched for transcription factors that are regulated by S100A2 and affect GLUT2 expression. Using the hTF target and JASPAR websites for prediction, BRD2, CTCF, ETS1, HNF1A, and CEBPB were identified as potential transcription factors for GLUT2 (Supplementary Fig. 4C). We first performed a preliminary screen by examining the mRNA expression of GLUT2 by overexpression of transcription factors. Overexpression of transcription factors BRD2 and CTCF in OSRC-2 cells did not result in a concomitant increase in GLUT2 expression (Supplementary Fig. 4,D). However, the mRNA level of GLUT2 increased after overexpressing ETS1, CEBPB, and HNF1A (Fig. 6B). Next, we wondered whether S100A2 might affect the regulation of GLUT2 by transcription factors through protein-protein interaction. To this end, we performed a co-immunoprecipitation experiment. The Co-IP experiment showed that S100A2 interacts with HNF1A, not ETS1 or CEBPB. (Fig. 6C and Supplementary Fig. 4E, F). Meanwhile, the interaction between S100A2 and HNF1A was confirmed by endogenous Co-IP assay in the 769-P cell line (Fig. 6D). Immunofluorescence experiments showed that S100A2 and HNF1A were significantly co-localized in the nucleus in clear renal cell carcinoma cells (Fig. 6E) and in HEK293T cells (Supplementary Fig. 4,G), supporting the interaction between S100A2 and HNF1A. HNF1A, hepatocyte nuclear factor 1 homeobox A, was initially discovered in the liver and subsequently verified to be expressed in several organs including the pancreas, kidney, and intestine [25]. As a transcription factor, HNF1A is involved in the regulation of a range of metabolism-related genes [26]. The luciferase reporter assay demonstrated that HNF1A enhanced the promoter activity of GLUT2 (Fig. 6F). To further verify the role of HNF1A in the regulation of GLUT2, we knocked down HNF1A in the 769-P cell line, and the GLUT2 mRNA and protein levels were knocked down (Fig. 6G). The correlation analysis confirmed a strong positive correlation between HNF1A and GLUT2 (Supplementary Fig. 4,H) and the JASPAR website predicted the binding site of HNF1A in the GLUT2 promoter (Supplementary Fig. 4,I).

To explore deeper into how S100A2 binds to HNF1A, we constructed four HNF1A mutants based on its structure, which contains a dimer domain, a DNA-binding domain, and a trans-activation domain (Fig. 6H). Co-immunoprecipitation results showed that the binding of S100A2 to HNF1A was disrupted only when the transactivation domain was absent, which indicated that S100A2 specifically interacted with the trans-activation domain of HNF1A (Fig. 6I). To establish a more comprehensive understanding of the S100A2/HNF1A complex, we analyzed the structures of HNF1A (AF-P20823-F1) and S100A2 (2RGI) from the Protein Data Bank database. Subsequently, we employed HDOCK to generate an interactive docking prediction of the HNF1A-S100A2 complex and symmetric multimers. The HNF1A dimerization domain model (aa 1–32) aligned well with the crystal structures available in the Protein Data Bank (Supplementary Fig. 4J). The predicted conformation, maintained by HNF1A, indicated the interaction with S100A2 in the transactive domain. Notably, the docking score was -218.95, where a more negative score indicates a more likely binding model. The confidence score of 0.7988 further supported a high likelihood of binding (Supplementary Fig. 4K).

Given that the transactivation domain of transcription factors can recruit co-factors and RNA polymerase II to enhance their transcriptional activity [27, 28], so we focused on RNA polymerase II. By Co-IP assay, we found that HNF1A could recruit more RNA polymerase II after S100A2 overexpression (Fig. 6J). In summary, these findings suggest that S100A2 promotes HNF1A to recruit more RNA polymerase II, thereby enhancing GLUT2 expression.

## Discussion

S100 family proteins have emerged as pivotal contributors to cancer development and progression [29]. The role of S100A2 in tumors has exhibited a dual nature, functioning as either a cancer promoter or a cancer suppressor through diverse molecular mechanisms. However, the specific mechanism underlying the action of S100A2 in ccRCC has remained elusive. As a significant subtype of renal cell carcinoma, ccRCC is characterized as a metabolic tumor, showcasing substantial intracellular reprogramming of glucose metabolism. In this study, our findings indicate a correlation between elevated S100A2 expression and poor prognosis in ccRCC patients. Consistent with the clinical data, our in vitro and in vivo experiments provide solid evidence that S100A2 plays a crucial role in promoting the metastasis and progression of ccRCC. We have elucidated a novel mechanism wherein S100A2 functions as a co-transcription factor. It binds to the transcription factor HNF1A within the nucleus, facilitating the recruitment of HNF1A to RNA polymerase II. This interaction activates the transcription of the target gene GLUT2, subsequently enhancing glucose uptake in renal cancer cells and promoting the glycolytic process. This intricate interplay between S100A2 and HNF1A sheds light on their collaborative role in regulating glucose metabolism in the context of ccRCC.

Regarding tumor metastasis, S100A2 plays a significant pro-metastatic role in ccRCC. Our study shows that S100A2 is markedly upregulated during the late stages of ccRCC progression, specifically during metastasis. As evidenced by clinical data, the analysis indicates that S100A2 is upregulated in the later stages of tumor metastasis. Additionally, in vivo and in vitro experiments support S100A2’s role in promoting metastasis. Overexpression of S100A2 significantly enhances the migratory and invasive capabilities of renal cell carcinoma cells, while its knockout inhibits these abilities. Therefore, S100A2’s pro-metastatic effects are evident.

Metabolic reprogramming is a defining feature of cancer [30]. While normal cells primarily rely on mitochondrial oxidative phosphorylation for ATP production, cancer cells, including those in ccRCC, favor aerobic glycolysis, a phenomenon known as the “Warburg effect” [31]. Many studies have found that tumor metastasis and invasion in carcinoma are associated with enhanced aerobic glycolysis [32]. The hypoxic nature of tumor tissues drives cancer cells to metastasize to other sites to secure increased energy and blood supply for their survival. Aerobic glycolysis contributes to tumor metastasis and invasion mainly through acidification of the extracellular environment, which is mediated by lactate and H + . This process involves several key mechanisms: (1) Low extracellular pH leads to the destruction of normal tissues through caspase-mediated or p53-dependent apoptosis [33] (2) Acidification of the extracellular matrix (ECM) promotes the secretion of proteolytic enzymes like cathepsin B or metalloproteinases, which assist in ECM degradation and facilitate metastasis [32]. (3) The immune suppression caused by low pH enables metastatic cancer cells to evade immune surveillance, resulting in persistent metastasis [34]. Thus, aerobic glycolysis is crucial in altering the extracellular structure and inducing immune suppression, which together make cancer cells more prone to metastasis and invasion. Despite being observed for nearly a century, the molecular drivers of this metabolic switch in cancer cells remain largely elusive.

Previous studies have reported that in colorectal and nasopharyngeal cancers, S100A2 promotes tumor progression by enhancing glycolysis through the PI3K-AKT pathway, which upregulates GLUT1 expression [35, 36]. Our study demonstrates that in ccRCC, S100A2 functions as a cofactor assisting the transcription factor HNF1A in promoting GLUT2 expression, thereby enhancing glycolysis and driving tumor progression. These different studies highlight a potential tumor-specific regulatory mechanism for S100A2 in glucose metabolism. In colorectal and nasopharyngeal cancers, the reliance on the PI3K-AKT-GLUT1 axis may reflect the metabolic demands or signaling dependencies unique to these cancer types. By contrast, in ccRCC, the S100A2-HNF1A-GLUT2 axis appears to play a predominant role, possibly due to the distinct metabolic landscape of renal cancer cells, which may favor GLUT2 for glucose uptake and utilization. Additionally, variations in the tumor microenvironment, such as oxygen availability, nutrient supply, or specific oncogenic mutations, could influence the choice of glucose transporters and pathways engaged by S100A2. It is also possible that S100A2’s function as a transcriptional cofactor is more prominent in ccRCC due to the differential expression or activity of interacting proteins like HNF1A in this cancer type. These findings underscore the complexity of S100A2-mediated glycolytic regulation and suggest that the role of S100A2 in cancer metabolism is highly context-dependent. Further investigations into the interplay between S100A2 and other metabolic regulators across different cancer types are necessary to uncover potential therapeutic targets.

In ccRCC, glycolytic metabolism is affected by a variety of factors, including alterations in glucose transporters [37–39]. GLUT2, a member of the glucose transporter family, is predominantly found in the pancreas, liver, and kidney [40]. The increased expression of GLUT2 can promote glucose uptake by tumor cells, enhance glycolysis, and promote tumor progression [41].In the present study, we uncovered that S100A2 enhances the transcriptional regulation of GLUT2 through its interaction with HNF1A, thereby boosting the ability of ccRCC cells to uptake glucose. This newfound understanding not only deepens our comprehension of S100A2’s role in ccRCC pathogenesis but also opens avenues for potential therapeutic strategies targeting S100A2-related dysregulation in ccRCC.

However, our study is limited, and the specific molecular mechanism of S100A2 in RNA polymerase II recruitment and whether S100A2 affects the transcription elongation process still need to be further explored. Our study acknowledges certain limitations in its clinical translational potential. Previous work in our lab has demonstrated that inhibiting the cancer-promoting effects of S100A2 in tumors by blocking its interaction with KPNA2 can be effective [13]. Therefore, blocking the interaction between S100A2 and HNF1A presents a promising therapeutic target for treating ccRCC. Further research is needed to validate these findings and explore the therapeutic implications fully.

## Conclusion

**Fig. 7:**
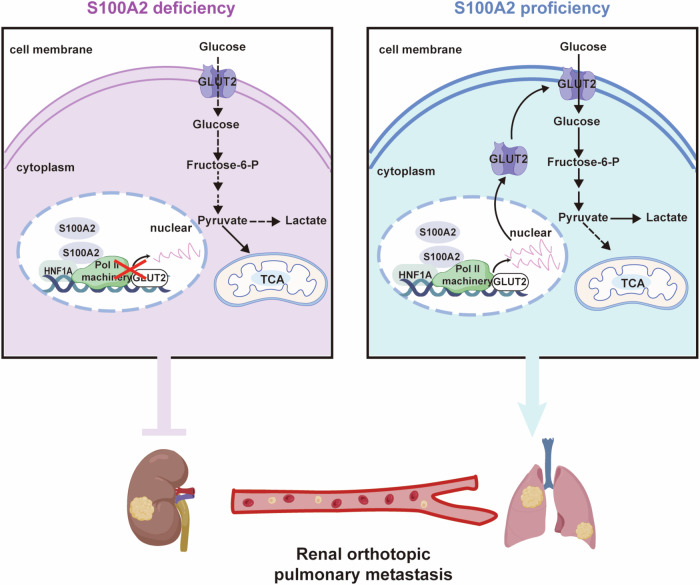
Schematic model of the mechanism of S100A2-HNF1A-GLUT2 axis in ccRCC metastasis. S100A2 plays a pivotal role in ccRCC by augmenting the transcriptional regulation of GLUT2 through its interaction with HNF1A. This interaction enhances the capacity of ccRCC cells to uptake glucose, ultimately amplifying glycolytic metabolism. The higher levels of glycolysis play a role in promoting distant metastasis in this scenario.

S100A2 enhances the transcription of GLUT2 through its interaction with HNF1A, thereby increasing the intracellular absorption and metabolism of glucose and promoting ccRCC metastasis (Fig. 7). S100A2 may serve as a potential therapeutic target for metastatic ccRCC in the future.

## Supplementary information

Supplemental material

Supplemental figure 1

Supplemental figure 2

Supplemental figure 3

Supplemental figure 4

original data

original data

## Data Availability

The accession number for RNA sequencing data is PRJNA1108977 and other data needed to evaluate the conclusions in the paper are present in the Supplementary Materials. Additional data related to this paper may be requested from the authors.
