# Supplementary material for: S100A2 promotes clear cell renal cell carcinoma tumor metastasis through regulating GLUT2 expression
Source: Cell Death Dis. 2025 Feb 27;16(1):135. doi: 10.1038/s41419-025-07418-1 (PMC11865524; doi:10.1038/s41419-025-07418-1)
Supplement: Supplementary file 1 — Supplemental material [file 41419_2025_7418_MOESM1_ESM.doc]

**Supplementary Information**

**S100A2 promotes clear cell renal cell carcinoma tumor metastasis through regulating GLUT2 expression**

**The file includes:**

Figure S1. High expression of S100A2 is associated with poor prognosis in ccRCC patients.

Figure S2. S100A2 promotes migration and invasion in vitro.

Figure S3. S100A2 promotes ccRCC migration by regulating the glycolysis pathway.

Figure S4. S100A2 overexpression increases GLUT2 expression by interacting with HNF1A.

Key resources tables

**Supplementary Figure Legends:**

**Figure S1. High expression of S100A2 is associated with poor prognosis in ccRCC patients.**

1. Kaplan-Meier curves of overall survival based on samples from TCGA ccRCC datasets stratified by S100A1, S100A3, S100A4, S100A5, S100A6, S100A8, S100A9, S100A10, S100A11 mRNA expression levels.
2. Kaplan-Meier curves of overall survival based on samples from ArrayExpress database stratified by S100A2 mRNA expression levels.

Statistical analyses were performed using the log-rank test, *P < 0.05, **P < 0.01, ***P < 0.001, ****P < 0.0001.

**Figure S2. S100A2 promotes migration and invasion in vitro.**

1. Real-time qPCR analysis of S100A2 mRNA expression in 786-O cells with knocking down of S100A2 by shRNA.
2. CCK-8 assays of the proliferation of S100A2-knockdown 786-O cells.
3. Colony formation assay of 786-O cells with knocking down of S100A2 by shRNA.
4. Real-time qPCR analysis of S100A2 mRNA expression in 769-P cells with knocking down of S100A2 by shRNA.
5. CCK-8 assays of the proliferation of S100A2-knockdown 769-P cells.
6. Colony formation assay of 769-P cells with knocking down of S100A2 by shRNA.
7. Alignment of sequencing results for validation of S100A2 knockout in 769-P cells with sgRNA.
8. CCK-8 assays of the proliferation in 769-P cells with knocking out of S100A2.
9. Colony formation assay of 769-P cells with knocking out of S100A2.
10. Western blot analysis of S100A2 protein knockout and re-overexpression in 769-P cells.
11. Transwell assay for evaluating the migration and invasion abilities of S100A2 protein knockout and re-overexpression in 769-P cells.
12. CCK-8 assays of the proliferation in S100A2-overexpression OSRC-2 cells.
13. CCK-8 assays of the proliferation in S100A2-overexpression ACHN cells.
14. Colony formation assay of S100A2-overexpression OSRC-2 cells.
15. Colony formation assay of S100A2-overexpression ACHN cells.
16. Western blot analysis of S100A2 protein overexpression in different levels in OSRC-2 cells.
17. Transwell assay for evaluating the migration and invasion abilities of S100A2 protein overexpression in different levels in OSRC-2 cells.
18. Western blot analysis of S100A2 protein overexpression and re-silencing in OSRC-2 cells.
19. Transwell assay for evaluating the migration and invasion abilities of S100A2 protein overexpression and resilencing in OSRC-2 cells.

The data are presented as the mean ± SD; statistical significance was assessed by one-way ANOVA (A, B, D, E, and H), two-way ANOVA (K, Q, and S), and unpaired t-test (L and M). *P < 0.05, **P < 0.01, ***P < 0.001, ****P < 0.0001.

**Figure S3. S100A2 promotes ccRCC migration by regulating the glycolysis pathway.**

1. RT-qPCR to determine the relative mRNA expression of the glycolysis pathway regulated by S100A2, including ENO2, GPI, NUP58, SLC37A1, PKLR, and PFKFB3.
2. Relative values of glucose in OSRC-2 EV and S100A2-OE cells.
3. ECAR assay for evaluating the glycolytic function of S100A2 protein knockout and re-overexpression in 769-P cells. The histograms on the right show the quantification of glycolysis, glycolytic capacity, and glycolytic reserve in the ECAR assays.
4. Glucose uptake analysis of S100A2 protein knockout and re-overexpression in 769-P cells.
5. Lactate production analysis of S100A2 protein knockout and re-overexpression in 769-P cells.
6. ECAR assay for evaluating the glycolytic function of S100A2 protein overexpression and re-silencing in OSRC-2 cells. The histograms on the right show the quantification of glycolysis, glycolytic capacity, and glycolytic reserve in the ECAR assays.
7. Glucose uptake analysis of S100A2 protein overexpression and knock down in OSRC-2 cells.
8. Lactate production analysis of S100A2 protein overexpression and knock down in OSRC-2 cells.
9. Transwell assay for evaluating the migration and invasion abilities of S100A2 protein overexpression with or without 2-DG treatment in OSRC-2 cells.

The data are presented as the mean ± SD; statistical significance was assessed by two-way ANOVA (A, C, F, and I), unpaired t-test (B), and one-way ANOVA (D, E, G, and H). *P < 0.05, **P < 0.01, ***P < 0.001, ****P < 0.0001.

**Figure S4. S100A2 overexpression increases GLUT2 expression by interacting with HNF1A.**

1. RT-qPCR analysis and western blot analysis of HIF-1α expression in S100A2-overexpression OSRC-2 cells.
2. RT-qPCR analysis and western blot analysis of GLUT2 expression in S100A2-overexpression ACHN cells.
3. Diagram showing transcription factors that bind the promoter region of GLUT2 identified in the JASPAR and hTF target databases.
4. RT-qPCR analysis of GLUT2, BRD2, and CTCF mRNA expression in BRD2-overexpression and CTCF-overexpression OSRC-2 cells.
5. Western blot analysis to detect IP of exogenous FLAG-tagged S100A2 and HA-tagged ETS1 by an anti-Flag antibody in HEK293T cells.
6. Western blot analysis to detect IP of exogenous FLAG-tagged S100A2 and HA-tagged CEBPB by an anti-Flag antibody in HEK293T cells.
7. Confocal microscopy and immunofluorescence staining of HEK293T revealed that S100A2 and HNF1A colocalize in the nucleus.
8. Correlation between GLUT2 and HNF1A mRNA levels in ccRCC.
9. Schematic of the putative HNF1A binding motif and relative score determined using JASPAR.
10. Alignment of the predicted HNF1A with the crystal structures for HNF1A dimerization domain (aa 2-32; PDB ID 2GYP, red). Among the regions, aa 91-181 are colored yellow, aa 203-279 are colored green, and aa 281-631 are colored purple.
11. Protein-protein docking results for S100A2 and HNF1A. The 3D structure of S100A2 is shown in surface format. The behind histogram shows the score results via the H-Dock website.

The data are presented as the mean ± SD; statistical significance was assessed by an unpaired t-test(A), and two-way ANOVA (B and D). *P < 0.05, **P < 0.01, ***P < 0.001, ****P < 0.0001.

**Key resources tables**

| **Reagents or Resource** | **Source** | **Identifier** |
| --- | --- | --- |
| **Antibodies** |  |  |
| Mouse monoclonal anti-FLAG® M2 | Sigma-Aldrich | Cat# F1804  （1：2000） |
| Rabbit monoclonal anti-HA-tag | Cell Signaling Technology | Cat# 3724S  （1：1000） |
| Mouse monoclonal anti-Beta Actin | Proteintech | Cat# 66009-1-Ig  （1：10000） |
| Goat monoclonal anti-S100A2 | R&D systems | Cat# AF4870  （1：500） |
| Rabbit monoclonal anti-S100A2 | Abcam | Cat# ab109494  （1：500） |
| Rabbit monoclonal anti-GLUT2 | Abclonal | Cat# A12307  （1：1000） |
| Rabbit monoclonal anti-ETS1 | Abcam | Cat# ab307672  （1：1000） |
| Rabbit monoclonal anti-HIF1α | Abcam | Cat# ab51608  （1：1000） |
| Rabbit monoclonal anti-RNA polymerase II | Abcam | Cat# ab193467  （1：1000） |
| Rabbit monoclonal anti-CEBPB | HUABIO | Cat# ET1610-9  （1：1000） |
| Alexa ® Fluor 546 Goat anti-Rabbit IgG (H + L) | Invitrogen | Cat# A-11010  （1：500） |
| Alexa ® Fluor Plus 647 Goat anti-Mouse IgG (H + L) | Invitrogen | Cat# A32728  （1：1000） |
| Rabbit monoclonal anti-HNF1A | Cell Signaling Technology | Cat# D7Z2Q  （1：1000） |
| Rabbit monoclonal anti-IgG | Cell Signaling Technology | Cat# 2729S  （1：1000） |
| IRDye® 800CW Goat-anti-Rabbit Antibody | LI-COR | Cat# 926-32211  （1：5000） |
| IRDye® 680CW Goat-anti-Mouse Antibody | LI-COR | Cat# 926-68070  （1：5000） |
| IRDye® 800CW Donley-anti-Goat Antibody | LI-COR | Cat# 926-32214  （1：5000） |
| **Bacterial** |  |  |
| DH5a | Tsingke | Cat# TSC-C14 |
| **Chemicals, Peptides, and Recombinant Proteins** | | |
| G 418 | Sigma | Cat# G5013 |
| Puromycin | Sigma | Cat# P8833-10MG |
| Trizol | Invitrogen | Cat# 15596018CN |
| LipoD293 | SignaGen | Cat# SL100668 |
| GenMute Reagent | SignaGen | Cat# SL100568 |
| Matrigel | Corning | Cat# 356234 |
| Fibronectin Purified Protein | Millipore | Cat# FC010-1MG |
| IP lysate | Beyotime | Cat# P0013J |
| RIPA lysis buffer | Fdbio Science | Cat# FD009 |
| Cocktail | MCE | Cat# HY-K0011 |
| PMSF | Beyotime | Cat# ST506 |
| ChamQ Universal SYBR qPCR Master Mix | Vazyme | Cat# Q711-02 |
| Mycoplasma Detection Kit | Yeasen | Cat# **40612ES** |
| HiScript II Q RT SuperMix | Vazyme | Cat# R223-01 |
| Hematoxylin | Sigma-Aldrich | CAT# H9627 |
| Eosin | Sigma-Aldrich | CAT# 230251 |
| Citrate buffer | ZSGB-BIO | CAT# ZLI-9065 |
| Goat anti-rabbit secondary antibody | ZSGB-BIO | CAT# PV6001 |
| 3,3'-diaminobenzidine (DAB) chromogen | ZSGB-BIO | CAT# PV8000 |
| 2-Deoxy-D-glucose | MCE | HY-13966 |
| 4,6-diamino-2-phenyl indole | Invitrogen | D1306 |
| **Critical Commercial Assays** | | |
| Dual-Luciferase Reporter Assay Kit | Promega | Cat#E1910 |
| BCA protein assay | ThermoFisher | Cat#23225 |
| ClonExpress-II One-Step Cloning Kit | Vazyme | Cat#C112 |
| CCK-8 | Biosharp | Cat#BS350A |
| Protein A/G immunoassay (coprecipitation) kit | Biolinkedin | Cat# IK-1004 |
| Anti-FLAG® M2 Magnetic Beads | Sigma | Cat#M8823 |
| Anti-HA Magnetic Beads | Sigma | Cat#SAE0197 |
| Glucose Assay Kit | Sigma | Cat#GAGO20 |
| Lactic Acid assay kit | Nanjingjiancheng | Cat#A019-2-1 |
| Glycolytic stress test kit | Agilent | Cat#103020-100 |
| **Mycoplasma Elimination Reagent** | Yeasen | Cat#40607ES |
| **Deposited Data** |  |  |
| Raw and analyzed data | This paper | NCBI BioProject ID:PRJNA1108977 |
| **Experimental Models:**  **Cell Lines** |  |  |
| 769-P |  | From ATCC |
| 786-O |  | From ATCC |
| OSRC-2 |  | From ATCC |
| ACHN |  | From ATCC |
| HEK293T |  | Chinese Academy of Sciences |
| **Experimental Models: Mice** |  |  |
| BALB/c nude | Shanghai SLAC Laboratory | www.slaccas.com |
| **Oligonucleotides** |  |  |
| **CRISPR gRNA sequences** |  |  |
| S100A2 KO gRNA1 | AGAGGGCGACAAGTTCAAGC | Cas9 |
| S100A2 KO gRNA2 | AAGGAGCTGCCCAGCTTTGT | Cas9 |
| **siRNA** |  |  |
| **Target** | **Sense** | **Antisense** |
| GLUT2-Homo-639 | CACGGGCAUUCUUAUUAGUTT | ACUAAUAAGAAUGCCCGUGTT |
| GLUT2-Homo-776 | CCAGAUACCUUUACAUCAATT | UUGAUGUAAAGGUAUCUGGTT |
| S100A2-Homo-423 | GACAAGUUCAAGCUGAGUATT | UACUCAGCUUGAACUUGUCTT |
| HNF1A-Homo-658 | GUCCCAACACCUCAACAAGTT | CUUGUUGAGGUGUUGGGACTT |
| siRNA Negative Control | UUCUCCGAACGUGUCACGUTT | ACGUGACACGUUCGGAGAATT |
| **qPCR Primer Sequences** |  |  |
| **Target** | **F** | **R** |
| S100A2 | CCAAGAGGGCGACAAGTT | TGATGAGTGCCAGGAAAA |
| ENO2 | CCGTAATCCCAGTGTGCTGT | CACATCGTTCCCCCAAGTCA |
| GPI | TACTCTTCAGGAGGTCCCCG | GATGAACAGGGAGGACTCGG |
| NUP58 | GGCTTTGAGGCGAGAGAAGT | CGTTCCGAAGGAGAAAACGC |
| SLC37A1 | GGACACCTTCTTTCCACGCT | GAGCCTCGGTCGCAGAATAA |
| PKLR | GTCCAGAGTCGGAAGTGGAG | ACCACTAGGGAGATGAGCCC |
| PFKFB3 | CCCGCTCATGAGACGCAATA | GCTGTTGATGCGAGGCTTTT |
| GLUT1 | CAGAAGGTGATCGAGGAGTTC | AGAGAAGGAGCCAATCATGCC |
| GLUT2 | AGCATCGAGTGAGCAGAAAG | GAAAATTGCTGAGCCACATGC |
| GLUT3 | AGTGTGCTTTAGCTTGAAAAGGT | TCAGTGAGAAATGGGACCCTG |
| HNF1A | CAGGTGATGAGCTACCAACCA | TTGCTAGGGTTCTTCTGCCTC |
| BRD2 | TAGCCCTCCTGGGAGTCTTG | GAGTCAGGCAAGTCTTTGCG |
| CTCF | ACGTCACATTCGCTCTCATACT | GGGTTCTCATGTGCCTTTTCA |
| CEBPB | AGAAGACCGTGGACAAGCAC | GCTTGAACAAGTTCCGCAGG |
| ETS1 | GCAGTGGACCAATCCAGCTAT | TTTGAATTCCCAGCCATCTCCT |
| HIF1α | GAACGTCGAAAAGAAAAGTCTCG | CCTTATCAAGATGCGAACTCACA |
| β-actin | GTCATTCCAAATATGAGATGCGT | GCTATCACCTCCCCTGTGTG |
| **Recombinant DNA** |  |  |
| **Vector Name** | **Vector** | **Application** |
| pLentiCRISPR v2-S100A2-KO#1 | pLentiCRISPR v2 | CRISPR/Cas9 |
| pLentiCRISPR v2-S100A2-KO#2 | pLentiCRISPR v2 | CRISPR/Cas9 |
| Flag-S100A2-OE | PCDH-CMV-MCS-EF1-CopGFP-T2A-puro | Overexpression and Co-Immunoprecipitation |
| pLKO.1-S100A2-sh#1 | pLKO.1 | Knockdown |
| pLKO.1-S100A2-sh#2 | pLKO.1 | Knockdown |
| HA-HNF1A-OE | pcDNA3.1(+)C-HA | Overexpression and Co-Immunoprecipitation |
| HA-BRD2-OE | pcDNA3.1(+)C-HA | Overexpression and Co-Immunoprecipitation |
| HA-CTCF-OE | pcDNA3.1(+)C-HA | Overexpression and Co-Immunoprecipitation |
| HA-CEBPB-OE | pcDNA3.1(+)C-HA | Overexpression and Co-Immunoprecipitation |
| HA-ETS1-OE | pcDNA3.1(+)C-HA | Overexpression and Co-Immunoprecipitation |
| HA-HNF1A-MUT1-OE | pcDNA3.1(+)C-HA | Co-Immunoprecipitation |
| HA-HNF1A-MUT2-OE | pcDNA3.1(+)C-HA | Co-Immunoprecipitation |
| HA-HNF1A-MUT3-OE | pcDNA3.1(+)C-HA | Co-Immunoprecipitation |
| HA-HNF1A-MUT4-OE | pcDNA3.1(+)C-HA | Co-Immunoprecipitation |
| **Software and Algorithms** |  |  |
| GraphPadPRISM | Open source | https://www.graphpad.com |
| SPSS Statistics 20 | Open source |  |
