# Supplementary figures and images for: S100A2 promotes clear cell renal cell carcinoma tumor metastasis through regulating GLUT2 expression
Source: Cell Death Dis. 2025 Feb 27;16(1):135. doi: 10.1038/s41419-025-07418-1 (PMC11865524; doi:10.1038/s41419-025-07418-1)

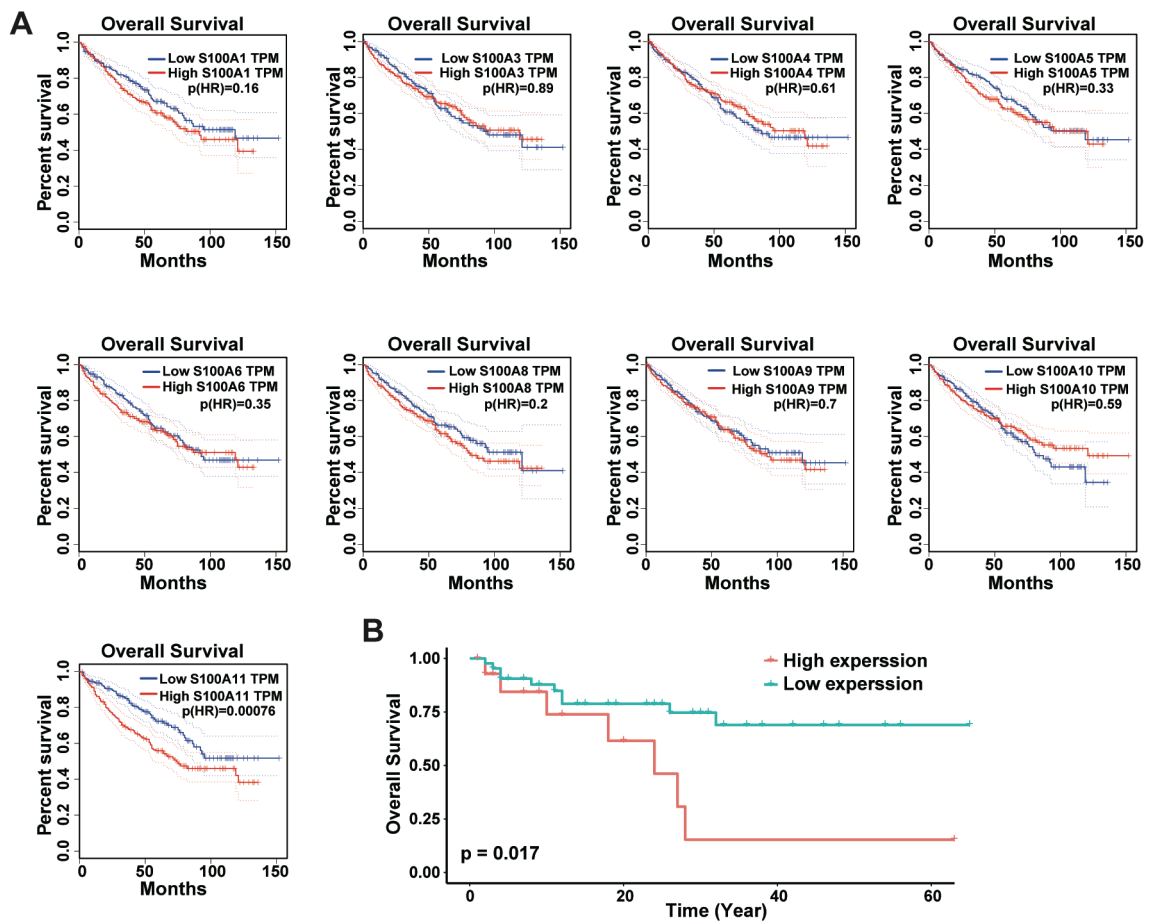

Supplement: Supplementary file 2 — Supplemental figure 1 [file 41419_2025_7418_MOESM2_ESM.pdf]

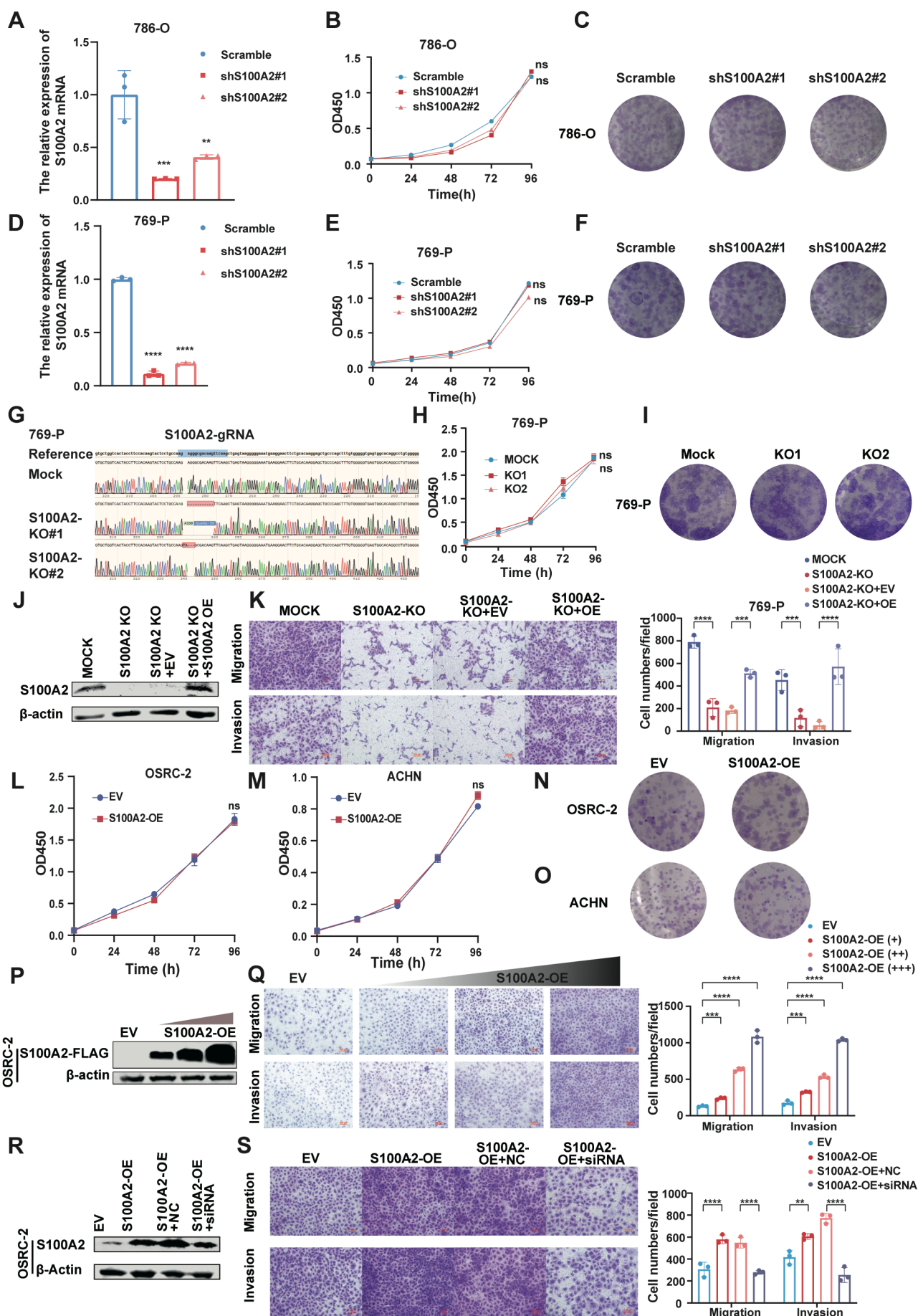

Supplement: Supplementary file 3 — Supplemental figure 2 [file 41419_2025_7418_MOESM3_ESM.pdf]

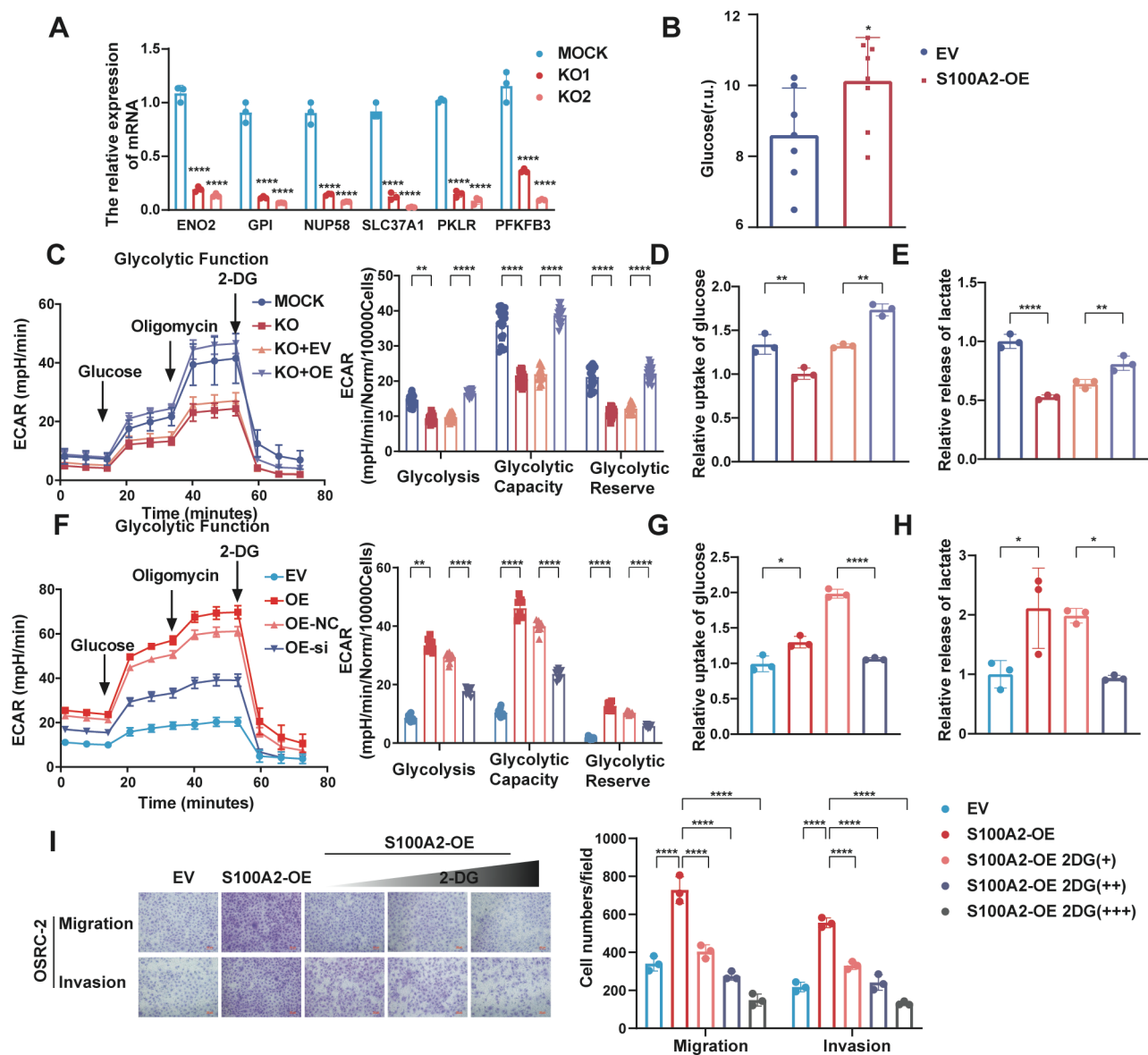

Supplement: Supplementary file 4 — Supplemental figure 3 [file 41419_2025_7418_MOESM4_ESM.pdf]

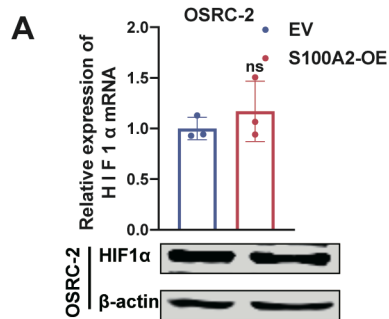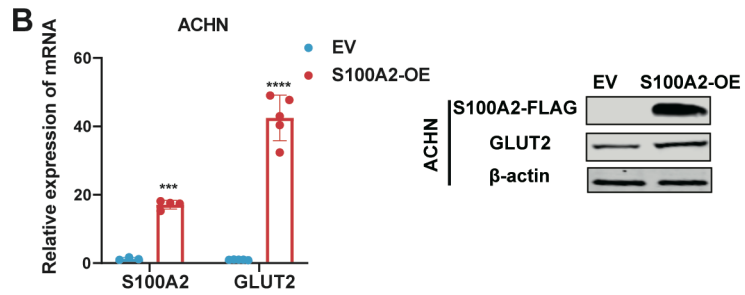

**C**

| hTF target | JASPAR |
|------------|--------|
| BRD2       | HNF1A  |
| CTCF       | CEBPB  |
| ETS1       | ETS1   |

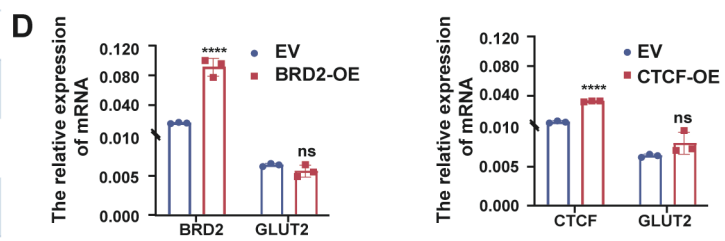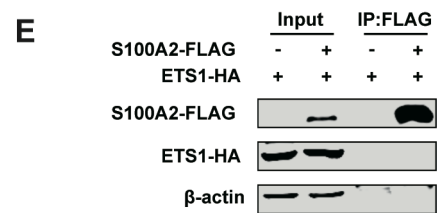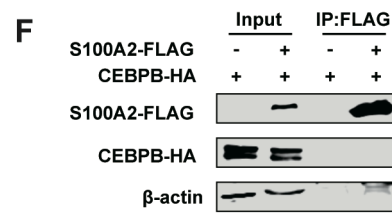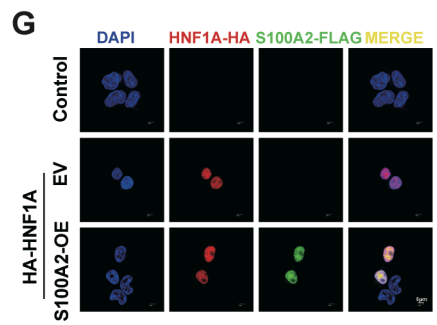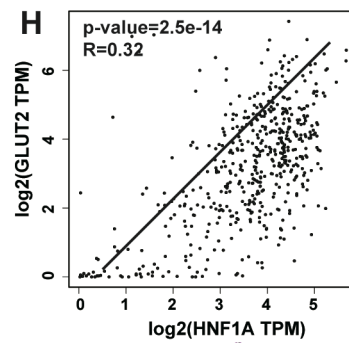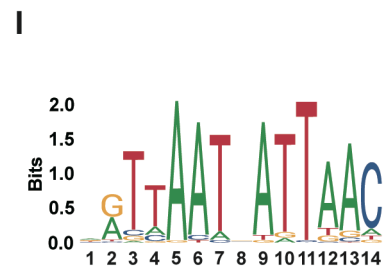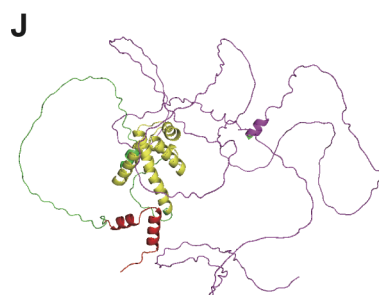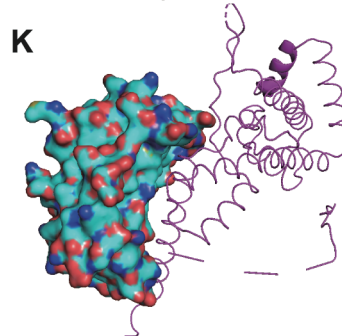

|                  |         |
|------------------|---------|
| Docking Score    | -218.95 |
| Confidence Score | 0.7988  |

Supplement: Supplementary file 5 — Supplemental figure 4 [file 41419_2025_7418_MOESM5_ESM.pdf]

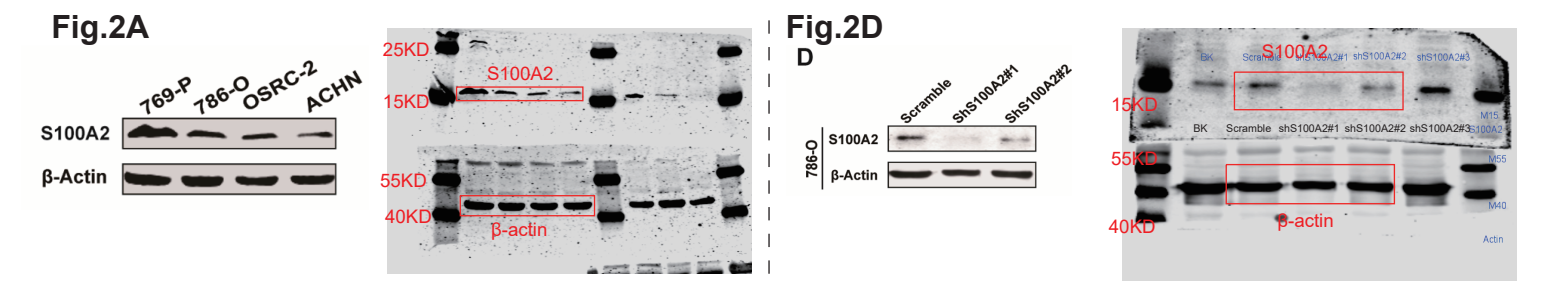

Supplement: Supplementary file 6 — original data [file 41419_2025_7418_MOESM6_ESM.pdf]
